# Examining types of Organizational Structure in Private Chartered Universities in Western Uganda and their Impact on Academic Staff Performance

**DOI:** 10.12688/f1000research.164151.3

**Published:** 2026-03-11

**Authors:** Turyamureeba silaji, Zulaihatu Lawal Bagiwa, Tukur Muhammad

**Affiliations:** 1 Department of Educational Foundation, Faculty of Education, Kampala International University, Western Campus, Uganda; 2 Department of Science, Faculty of Education, Kampala International University, Bushenyi, Western Campus, Uganda

**Keywords:** Organizational Structure; Academic Staff Performance; Private Universities; Uganda

## Abstract

**Background:**

This study examined organisational structure dimensions in privately chartered universities in Western Uganda and how they influence academic staff performance. Guided by Administrative Management principles and interpreted through Expectancy Theory, the study explains how institutional arrangements shape staff behaviour and performance outcomes rather than testing motivational constructs directly.

**Method:**

A convergent parallel mixed-methods design was employed, integrating quantitative and qualitative data collected concurrently. Quantitative data were obtained from 186 academic staff using structured questionnaires, while qualitative insights were gathered from 10 academic deans through in-depth interviews. Quantitative data were analysed using descriptive statistics, correlation, and regression, whereas qualitative data were analysed thematically, and the findings were merged during interpretation.

**Results:**

Findings revealed dominant mechanistic structural dimensions characterised by centralised decision-making and formalised procedures. A significant positive relationship was observed between organisational structure dimensions and academic staff performance (r = 0.512, p < 0.01), with organisational structure explaining 26.2% of the variance in performance (R ^2^ = 0.262, F(1,184) = 65.46, p < 0.001). However, qualitative findings indicated that excessive centralisation and bureaucratic control limited academic autonomy, collaboration, and innovation, resulting in performance that emphasised compliance rather than scholarly engagement.

**Conclusion:**

The study concludes that organisational structure influences academic staff performance but primarily promotes regulated performance rather than developmental academic productivity. Improving performance requires balancing structural coordination with participatory governance and supportive institutional practices that enhance professional motivation and engagement.

## Introduction

Bolman and Deal’s (2017) four-frame model has been empirically shown to influence organizational effectiveness in academic settings. Studies applying their framework demonstrate that universities employing a balanced approach across structural, human resource, political, and symbolic frames report higher faculty satisfaction, improved collaboration, and enhanced performance outcomes. For example, institutions that integrate structural clarity with supportive human resource practices and inclusive decision-making tend to foster better academic staff engagement and productivity. This empirical evidence underscores the importance of multifaceted organizational designs in optimizing academic staff performance.

Organizational structure plays a critical role in shaping the internal operations of education institutions ( Akinyele & Fasogbon, 2019). In private universities, the type of organizational structure adopted directly influences academic staff performance in terms of teaching, research, and administration. The structure defines authority lines, communication flows, decision-making processes, and division of labor, all of which have a bearing on staff efficiency and productivity. In Western Uganda, private universities have adopted various organizational structures, ranging from hierarchical to functional and, more recently, matrix-based models.

This study seeks to determine the types of organizational structures in use within privately chartered universities in Western Uganda and explore how these structures impact academic staff performance. Understanding the dominant structures and their effects on staff will help inform strategies for improving performance and fostering academic excellence.

Organisational structure defines the formal framework through which institutions coordinate activities, allocate authority, and achieve strategic objectives. In universities, structural configurations determine how academic and administrative roles interrelate, influencing efficiency, innovation, and staff motivation. In Uganda, private chartered universities operate under diverse structures some highly centralised, others more decentralised reflecting variations in ownership, governance, and institutional culture.

This study draws on Henri Fayol’s Administrative Management Theory, which emphasises division of work, authority, unity of command, and scalar chains as foundations for organisational efficiency. Complementarily, Vroom’s Expectancy Theory posits that performance is shaped by the perceived relationship between effort, performance, and reward. Integrating these frameworks provides a dual lens: Fayol explains how structural clarity shapes control and accountability, while Vroom elucidates how structure affects motivation and performance expectancy.

Despite increasing scholarly attention ( Mugizi et al., 2019d; Rwothumio & Amwine, 2021a; Silaji et al., 2025), gaps remain regarding how structural configurations in private universities influence academic staff productivity, particularly within Uganda’s expanding higher education sector. This study therefore investigates the relationship between organisational structure and academic staff performance in private chartered universities in Western Uganda. In this study, Expectancy Theory is used as an interpretative lens rather than a directly measured theoretical construct. The study did not operationalise the individual components of expectancy (expectancy beliefs, instrumentality, and valence) as empirical variables in either the quantitative or qualitative instruments. Instead, the theory provides an explanatory basis for understanding behavioural responses of academic staff to organisational practices. Specifically, organisational structure and performance monitoring were assumed to influence staff effort, persistence, and work engagement through perceived links between performance expectations and institutional outcomes. Thus, Expectancy Theory was employed to interpret how institutional arrangements shape motivation and performance behaviour, rather than to test the theory empirically.

The study does not categorise universities into discrete structural types; rather, it examines the extent to which specific organisational structure dimensions are present within institutions. The constructs measured reflect structural characteristics such as centralisation, formalisation, hierarchy, and participation in decision-making. Therefore, the term “types of structures” is used conceptually to describe dominant structural tendencies, not mutually exclusive organisational categories. Empirically, the analysis evaluates the configuration and strength of structural dimensions instead of classifying universities into predefined structural typologies. Consequently, the findings should be interpreted as identifying prevailing structural orientations rather than assigning institutions to fixed structural types.

## Literature review

Grounded in Henri Fayol’s Administrative Management Theory, as introduced by Fayol (1949), and Vroom’s Expectancy Theory, developed by Vroom (1964), this study integrates structural and motivational perspectives to explore the impact of institutional design on academic operations. Fayol’s theory emphasizes principles such as specialization, authority, and clear hierarchical structures, while Vroom’s model links motivation to expected outcomes, suggesting that organizational structure can directly influence staff motivation and performance. Additionally, Clegg et al. (2016) argue that organizational structures are not merely administrative tools but are embedded in power dynamics and cultural contexts that influence how staff engage with their institutions. By combining these theoretical lenses, the study offers a comprehensive framework for understanding how the structural design of universities affects academic staff effectiveness.

Several scholars, including Atwebembeire et al. (2018a, b), Malunda (2019), Birungi et al. (2021), Emuron et al. (2022), Adyanga et al. (2022), Kamanzi (n.d.), and Kansiime and Singh (2023), identified persistent gaps in teaching, research output, and evaluation criteria—factors crucial to the sustainability of private universities. While these studies employed various research designs, they primarily relied on quantitative approaches to assess academic staff performance. The current study addressed this methodological gap by adopting a mixed-methods research design, enabling a more comprehensive analysis of the issues.

Similar findings were reported by Mugizi, Silaji, and Abba (2018), who emphasized that the structure and governance of institutions significantly affect staff morale and commitment. Their study contributed to the understanding of how centralization and rigid control mechanisms often hinder staff performance.

Likewise, Mugizi et al. (2019a) highlighted that internal performance mechanisms, particularly those involving feedback and recognition, were inadequately implemented in private institutions—reinforcing the need for this study’s focus on performance monitoring. Also Performance monitoring enhances accountability according to Mugizi et al. (2019b).

According to Nuwatuhaire and Turyamureeba (2019), private universities in Uganda often struggle with aligning organizational structure and staff performance frameworks, which has led to inconsistencies in institutional output and staff engagement.

Mugizi et al. (2019c) underscored the urgent need for performance evaluation systems that align with strategic goals and promote academic excellence. Their work supported the rationale for this study’s investigation into the influence of performance monitoring.

Additionally, Rwothumio and Amwine (2021a) noted that management practices, especially those related to workload distribution and staff appraisal systems, had a direct impact on staff satisfaction and output in private universities. Comparable results were observed by Kabwe (2024), who found that private university staff in Zambia viewed performance appraisals as inconsistent and lacking developmental focus. Which is also consistent with Kimanje (2021), who reported that academic staff in Ugandan private universities perceived performance appraisal systems as irregular and largely administrative.

Turyamureeba (2019) also examined staff evaluation methods and found them to be sporadic and lacking transparency—an observation that informed this study’s emphasis on performance monitoring mechanisms.

Although Marisa and Oigo (2018) explored organizational structures in East African private universities, their findings remained largely descriptive and did not fully address the connection between structural design and academic staff performance, leaving a gap that the present study aimed to fill.

Organizational structures in higher education institutions vary widely based on the institution’s size, culture, and mission. According to Mintzberg (1979), universities typically adopt several organizational structures such as:

**Hierarchical Structure:** This traditional model emphasizes a clear chain of command, with decision-making authority concentrated at the top of the hierarchy. This is commonly found in institutions where control and accountability are prioritized ( Weber, 1947). Rwothumio et al. (2021a, 2021b) note that public universities implement structures and performance appraisal systems that play a critical role in shaping staff outcomes.

**Functional Structure:** In model, the organization is divided into specialized units or departments like teaching, research, and administration ( Burton & Obel, 2018a). It facilitates expertise development, but can lead to siloed work environments and communication breakdowns ( Lawrence & Lorsch, 1967). According to Burton and Obel (2018a), strategic alignment within an organization is achieved when its structure supports its goals. In the context of universities, Burton and Obel (2018b) emphasized that an appropriate organizational framework enhances decision-making and innovation. Furthermore, Burton and Obel (2018c) argued that the fit between structure and coordination is essential for performance optimization in complex institutional settings.

The matrix structure, which combines elements of both hierarchical and functional models to enhance flexibility and cross-departmental collaboration, has been widely discussed by scholars such as Galbraith (1973), who introduced the concept, and Burton and Obel (2018b), who elaborated on its application in complex organizational environments.

In Uganda, research by Silaji et al. (2025) indicates that private universities tend to adopt hierarchical or functional models, often influenced by the need for clear administrative control and specialization in teaching and research. However, there is an increasing trend toward adopting more flexible decentralized models in response to the growing demand for interdisciplinary collaboration and innovation.

Posselt, Hernandez, Villarreal, Rodgers, and Irwin (2020) conducted a study on Collegiality, Collaboration, and Hierarchy in Academic Decision-Making. To investigate the impact of collegial structures on academic decision-making. This study focused on how distributed authority among academic units influences collaboration, governance, and staff involvement. It was hypothesized that collegial structures positively impact faculty engagement and decision-making efficiency guided by the Collegial Model of Governance. This study employed a cross-sectional survey design and a quantitative approach using descriptive statistics and regression analysis to analyze the data. The findings revealed that institutions with collegial structures experienced a 25% increase in faculty satisfaction and a 15% improvement in collaborative research output. The study concludes that collegial models foster a collaborative environment that enhances academic performance and staff morale. It recommends that universities adopt shared governance models to boost institutional performance and faculty commitment. This research supports Bell et al. (2018) findings regarding the benefits of shared governance in improving academic staff performance.

Koigi, Maunganidze, and Mutambara (2018) conducted a study titled the Effect of Divisional Organizational Structure on Staff Academic Performance in Private Universities. Their research hypothesized that divisional structures enhance staff performance and operational efficiency compared to other organizational structures, guided by Divisional Structure Theory. The research, which focused on the academic performance of staff in private universities, used a comparative study design with a quantitative approach and employed ANOVA and performance metric analysis. The findings revealed that universities with divisional structures saw a 20% increase in staff performance ratings and 30% improvement in operational efficiency. The study recommends that private universities adopt divisional structures to improve performance and manage diverse academic programs effectively. These findings align with those of Koigi et al. (2018), who highlight the positive impacts of divisional structures on operational outcomes.

Building on this, Sakthivel and Raju (2020) explore the effects of matrix structures on innovation and staff performance in their study. Their study hypothesized that matrix structures foster innovation but introduce challenges related to role ambiguity and communication guided by Matrix Management Theory. The case study used a mixed-methods approach, incorporating both qualitative and statistical techniques to analyze the data. The findings showed that matrix structures led to a 22% increase in innovation outputs and an 18% improvement in staff performance, although 30% of the participants reported role conflicts. The study recommends implementing support systems to address communication issues and role conflicts inherent in matrix structures. This study builds on Sakthivel and Raju’s (2020) findings regarding the dual effects of matrix structures on performance and innovation.

In contrast, Dedahanov, Rhee, and Yoon (2017) investigated the impact of bureaucratic structures on staff efficiency and decision making. They hypothesized that bureaucratic structures decrease staff efficiency and decision-making speed, based on Bureaucratic Management Theory. The study used a quantitative survey design and approach with regression analysis and efficiency metrics for data analysis. The findings revealed a 15% decrease in staff efficiency and a 20% increase in decision-making time, with lower staff morale compared with more flexible structures. The study recommends introducing flexibility within bureaucratic structures to improve staff efficiency and morale. This study extends Dedahanov et al. (2017) by highlighting the limitations of bureaucratic structures in staff engagement.

Similarly, Ndirangu and Udoto (2021) assessed the impact of flat organizational structures on staff engagement and performance in their study. They hypothesized that flat structures increase staff engagement and performance, but may pose challenges in larger institutions, guided by the Flat Organization Theory. This survey-based study used a quantitative approach with statistical analysis and engagement metrics. Their findings showed a 28% increase in staff engagement and a 17% improvement in performance with flat structures, although larger institutions faced coordination challenges. The study recommends using flat structures in smaller units and considering hybrid models for larger institutions to address these coordination issues. This research supports Ndirangu and Udoto’s (2021) findings regarding the effectiveness of flat structures in enhancing engagement and performance.

Jameel and Ahmad (2020) explored the impact of leadership styles (LS) on educational institutions in developing countries, a topic that has not been extensively studied. Their research focused on examining how leadership styles affect academic staff performance (ASP) in Iraq. We hypothesized that both transformational leadership (TFL) and transactional leadership (TSL) would influence ASP. Additionally, job satisfaction (JS) was proposed as a mediating factor between LS, TFL, TSL, and ASP. The study surveyed 297 academic staff members from nine universities in Baghdad using a stratified sampling method. The results indicated that leadership styles, such as transformational leadership (TFL) and transactional leadership (TSL), would significantly affect ASP, with JS partially mediating this relationship. It is recommended that decision-makers emphasize TFL and work to enhance JS among academic staff.

Siddiqui (2022) conducted a study to determine the impact of a firm’s organizational structure on performance by evaluating both financial and non-financial aspects. The research involved a systematic review of the literature, analyzing 35 papers from selected management, finance, and other relevant journals. Data and conclusions from several other geographic regions, industries, and company sizes were incorporated into the final publications. A variety of organizational structures, including those comprising hybrid internal systems, were examined. Similarly, the performance analysis includes both objective and subjective metrics. The review’s findings were divided into three categories: the organizational structure’s favorable impact on company performance, its partial impact on firm performance, and its absence from the picture altogether. The results of the evaluated papers are presented in a table with pertinent data. Future study proposals were provided since there was no clear connection between business structure and performance.

According to Mugizi et al. (2019c), organizational structure is a representation of the network of task and authority relationships that controls how academic staff use resources to accomplish organizational goals. Centralization boosts decision-making at higher hierarchical levels of an organization while decreasing employee engagement in decision-making. Inter-organizational division, which includes specialization, classes of labor, and layer numbers in the organizational hierarchy, is referred to as complexity ( Mugizi & Dahiru Abba, 2018). The number of professional specializations present in an organization and the time required for training are considered complexity or specialization ( Atwebembeire, 2018a). The degree of specialization is differentiated by person and task specializations. The complexity of an organization increases with the number of person specialists and the length of training necessary to achieve person specialization ( Zhang & Liu, 2017; Atwebembeire, 2018a). Departments with integrated or functionally specialized staff can be found in complex or specialized structures. High levels of horizontal integration suggest that employees within departments are well integrated in terms of their work, skills, and training. In contrast, low levels of horizontal integration imply that departments and staff members are more functionally specialized ( Mugizi & Nuwatuhaire, 2019). Gao et al. (2019) claimed that perceived responsibility and role involvement, and subsequently organizational commitment, may be affected by a decision-making organizational structure that encourages effective staff communication that keeps the individual informed about crucial organizational components.

Different scholars have argued that systems of education can be categorized as decentralized or centralized, in addition to variations in uniformity. While decentralized education systems allow local governments to centralize educational systems, centralized education systems would have education financing consolidated in a country across the whole educational system with minimal local autonomy and manage university funds for both public and private universities ( Broer, Bai, & Fonseca, 2019). Centralization often leads to the standardization of curriculum, instruction, and central exams in an educational system, because it lessens the influence of a student’s familial background. This can be beneficial for reducing disparities ( Broer et al., 2019). However, high levels of decentralization can lead to greater educational gaps, particularly when funding levels are dictated by local conditions ( Hertwig & Engel, 2021). The once-controlled Swedish educational system has undergone a significant transformation into one that is extremely decentralized and unregulated, with an increase in the number of independent universities and parental control over their children’s education. Sweden’s educational system was centralized until the adoption of extensive reforms in the early 1990s; for example, see Atwebembeire (2018b). Researchers have concurrently evaluated the multilevel impacts of SES on reading achievement in Sweden using data from the 1991 IEA Reading Literacy Study and the 1991–2001 PIRLS. There is evidence that the SES effect has expanded over time since between-university inequalities were greater in 2001 than in 1991 ( Broer, Bai & Fonseca, 2019).

Although the majority of private universities in Uganda are for-profit organizations, there is little evidence that they adhere to a specific form of governance. The charter of the particular institution is required to outline the membership, authority, and responsibilities of such a body ( Sims, Lundie, Titus, & Govender, 2023). The four private universities included in this study have nearly identical governance structures, with some differences at the top depending on the ownership of the institution. While UNIK and UNIL are held by individual private investors, UNIN and ASUL, respectively, two of the four institutions are owned by the church of Uganda dioceses in the Buganda and Lango sub-regions, respectively. A university council (which controls the day-to-day operations of the institution), a senate (which examines policy), university committees, and many departments and faculties are at the top of each university. The nomination of senior administrators, including the chancellor, vice chancellor, council chairperson, and council members, is the responsibility of boards of trustees and/or directors. Thus, there was a power concentration at the top. As they are not represented, professional staff, administrators, faculty, and students are excluded from the process ( Ochwa-Echel, 2016).

The four most commonly discussed subdimensions of organizational structure are explained in more detail as follows: level of formalization, number of hierarchical levels, degree of horizontal integration, and concentration of power ( Berkovich, 2023). Formalization is assessed based on the number of rules and procedures given to staff, which may inhibit rather than promote creative, independent work, and learning. According to the organization theory literature, a mechanistic structure is connected to a high degree of formalization, whereas an organic structure is linked to a low level of formalization. Flexible work regulations promote creativity, but high degrees of formalization are believed to have the opposite impact, according to the literature on innovation ( Berkovich, 2023), size of the hierarchy in layers. The number of layers determines how many levels of management an organization has in comparison with how few ( Berkovich, 2023). According to ( Berkovich, 2023), organic organizations have a minimal hierarchy. The assumption in the literature on innovation is that hierarchical levels lengthen the links between channels, making it more difficult to communicate between levels and reducing the flow of innovative ideas ( Christopher, 2020).

The degree of horizontal integration is assessed based on the extent to which departments and employees are functionally specialized versus those who are well integrated in terms of their work, skills, and training (high level of horizontal integration) ( Laanpere, 2019; Aderibigbe, 2018).

To execute tasks in a sequential, coordinated manner, organizations often separate functional departments in line with the concept of division of labor. To respond to evolving environments and deliver value to customers, postmodern organizations have structured their workforce into autonomous work teams, cross-functional teams, and task forces. Because staff regularly obtain cross-training to better understand the entire process and adapt to customers’ changing demands, organic organizations have high degrees of horizontal integration ( Laanpere, 2019).

Decisiveness center. According to Kyatuha et al. (2016), when employing the control model of management, companies prioritize managerial prerogatives and positioning power, while also dispersing status symbols to support the hierarchy. The level of decision-making at each level of the organizational hierarchy is known as the locus of decision-making.

Decision-making should be delegated to organizations operating in ambiguous environments so that staff members can swiftly adjust to changing circumstances and provide value to customers ( Laanpere, 2019). Decision-makers can use management toolkits called monitoring systems.

To monitor the development and demonstrate the effects of a particular program or project. Over time, decision-making toolkits assist companies in determining the success, failure, relevance, effectiveness, and efficiency ( Christopher, 2020).

To perform successfully and efficiently and produce the necessary outcomes, monitoring and evaluation systems require 12 essential components ( Christopher, 2020). Administrative support refers to universities’ effectiveness in motivating lecturers, students’ discipline, university curriculum, instructional methodologies, and changing the university environment ( Mugizi & Nuwatuhaire, 2019). Effective administrative support plays an important role in university leadership, student discipline, academic work, guidance and counseling practices, and four dimensions: a vision of building a university, the development of goals and priorities, motivating staff, and the development of a collaborative university culture.

Mugizi, Nuwatuhaire, and Turyamureeba (2019b) carried out a study highlighting the critical role of administrative support in the survival and success of universities, particularly in influencing academic staff performance. Their findings indicated that effective administrative support significantly enhances lecturers’ efficiency and effectiveness, leading to improved performance outcomes. Conversely, the absence of administrative support has been linked to high turnover rates, with lecturers leaving the teaching profession because of unmet needs for assistance. The study also found that administrative support is a key motivator for lecturers in private universities, reinforcing their role in sustaining academic staff morale. Similar findings were reported in investigations conducted by public universities in New York, where administrative support was found to play a crucial role in teacher retention. Qualitative studies further support these findings, demonstrating that when lecturers feel neglected by the university administration, they often turn to alternative occupations, such as farming or boda riding, as a means of livelihood ( Carver & Darlin, 2019). Similally, Mugizi, Nuwatuhaire, and Turyamureeba (2019b) also empirically demonstrated that university leadership significantly impacts lecturers’ job satisfaction, which in turn positively affects both student and staff performance. Their research aligns with multiple studies indicating that strong administrative support is directly associated with higher levels of job satisfaction, resulting in a longer retention of academic staff. Atwebembeire and Malunda (2019) discovered that poor working conditions, such as insufficient administrative support, inadequate basic amenities, subpar facilities, student misbehavior, lack of motivation, and ineffective decision-making processes, lead to lecturers’ decisions to resign from their positions. Universities offering favorable working conditions, such as housing, allowances, secure environments, supportive administration, and fair compensation, were found to retain and attract high-quality lecturers. These improved conditions also foster increased motivation and performance among lecturers, ultimately benefiting students by enhancing the quality of the education delivered. Large class sizes may disrupt students’ attention, making both teaching and learning difficult ( Geiger & Pivovarova, 2018).

Research has shown that’cturersthe working conditions are crucial to their job satisfaction, regardless of their workplace. For example, the salaries of lecturers tend to be very low compared to those earned by qualified individuals in other professions, regardless of the type or location of the university. The working conditions for lecturers, such as job security and university safety, vary widely from university to university. In addition to lecturers’ working conditions, there is a need to understand the types of universities that tend to have desirable conditions to attract both students and lecturers. If working conditions are difficult and equity cannot be attained, the characteristics of students should be handled ( Geiger & Pivovarova, 2018). In addition, interpersonal relationships and good staff relationships are paramount, as they promote both social and professional harmony among lecturers and other members of the university. The social and professional relationships of lecturers create teamwork that enables universities to achieve their goals, thus surviving in this competitive world. Lecturers feel well when they work together in handling their responsibilities towards improving university objectives and students’ learning, thereby enticing lecturers to stay in universities for a long time ( Mugizi & Nuwatuhaire, 2019).

Work engagement is a motivator that boosts lecturers, as evidenced by their dedication, vigor, and absorption. Such lecturers enjoy problems with ease, exhibit mental resilience, are always patient, and engage in their usual activities. While studies of lecturers’ job involvement in education indicated that instructors demonstrated a lot of patience, satisfaction, monitoring, and successful teaching, research on work engagement has been undertaken in business settings ( Musenze et al., 2013). Lecturer work engagement can partly be influenced by university managers, such as deans, goods, and monitoring heads, among others. This results in proactive work behaviors that promote seriousness among staff members to increase service delivery. If university managers fail to motivate their lecturers, the reverse is true. According to the work engagement model, workplace resources, including materials and demands, impact lecturers’ professional development. In this case, job resources are the organizational, social, psychological, and physical components that lower the job demands, enhance lecturers’ abilities to carry out their tasks, and ultimately aid universities in achieving their goals. Additionally, they support personal development, growth, and learning ( Mugizi, Rwothumio & Amwine, 2021).

Similarly, for managers, job resources, including autonomy, respect, performance evaluation, encouragement of coworkers, and supervisory coaching, boost workplace engagement. The quality of their interactions with their peers in the teaching profession is a job resource. This could come from social and interpersonal ties, organizational structures, and the university system. Other elements, such as personal resources, can contribute to increased job engagement. Self-efficacy, optimism, and unique personalities, which can be molded to enhance work performance, are examples of personal resources. For example, academic affairs or an educational manager’s personal resources may include the control of educational activities, university roles, and personal experience. Communication among staff is vital as it encourages different ideas with other members of the community using an assortment of methods, such as expressions, gestures or signs, voice tone, facial expression, and body posture ( Kasule, Mugizi & Rwothumio, 2021).

The specialization of cooperative parties who carry out particular duties and roles is known as the division of labor ( Cooper & Gubler 2020). In ancient Sumerian (Mesopotamian) society, the idea and practice of division of labor was evident. In some places, employment distribution is accompanied by an uptick in trade and economic interdependence ( Cooper & Gubler 2020). Again, the division of labor typically boosts both producer and individual worker productivity, in addition to trade and economic interdependence. For instance, in a university context, the division of labor results in better coverage of tasks because different individuals can manage multiple projects and programs within a predetermined time limit. Each individual or family in such a complex society concentrates on producing only a small number of unique goods or services (or perhaps just a small portion of a larger good or service) and then purchasing all other desired goods or services from the production of other specialists through reciprocal exchange (or, in non-market economies, perhaps through coercive or customary transfer). Many universities and departments seek to cultivate certain capabilities in students, enabling them to graduate with the abilities required for a certain career ( Burton & Obel, 2018b).

Staff members participate in a variety of tasks at universities to guarantee that services are available to students and the general success of the institution. Although these activities may vary in several aspects, they are socially connected ( Kyatuha et al., 2016). Within an institution, there is a complex and dynamic interchange of collaboration and communication that has the potential to be contentious. The specialization of workers at universities is the core economic tactic utilized to provide basic community needs for health, food, shelter, and education, despite the contentious nature of this connection ( Mugizi, Nuwatuhaire & Turyamureeba, 2019b). The division of labor in higher institutions is caused by a variety of factors, some of which are neutral gender and others that are biased. Administration, planning, and teaching were assigned to the staff members. These are not necessarily products of talent based on comparative advantage, but rather of specialization. Other factors affecting labor division include the sharing of tasks among people based on descent, kinship, education, status, culture, marriage, and age, which are more prevalent in rural civilizations ( Belbin & Brown, 2022).

Similarly, it is a system of division of labor in which multiple tasks are carried out simultaneously by various people. Sometimes, the division of labor can be used in the context of economics; in contemporary society, it refers to all factors of production and extends beyond economics. The three types of division of labor ( Marmolejo & Groccia, 2022). The ability of an organization’s academic staff to bargain has a significant impact on the division of labor. The foundation of group decision making in negotiations is bargaining. Threats and promises that must be carried out outside the assembly are the primary tools used by negotiating players to increase their returns. Credibility rises in direct proportion to how appealing actors’ best available alternatives to an agreement are. Therefore, it is reasonable to assume that the outcome of a negotiation, or the distribution of profits, largely reflects the power dynamics between parties ( Blau, 2021).

Related to the above, for an organization to function well and foster a sense of cooperation and effectiveness, the right individuals must be in the proper positions of authority, carrying out the correct tasks. Therefore, this structure can be described as a hierarchy of interconnected positions, teams, and authorities. There is no set organizational structure, but the majority of businesses and government agencies use the structure of a Christmas tree, with the top branch (for instance, the deputy vice chancellors, vice chancellors, deans, and directors) and larger branches at the execution levels. Despite some claims, the organizational mission and objectives can be thought of as a single trunk that supports the branches of a tree, just as the top branches are supported by lower branches. Every branch of the tree serves a distinct purpose in the decision making process. The system exists, performs productively, has balance, and can achieve its goals when all its components work together ( Rwothumio et al., 2021a).

Again, Organizational and innovation studies contain the majority of the work on organizational structure. Most studies have indicated that organizational structure has many different aspects. A mechanical dichotomy versus an organic dichotomy is a traditional illustration of organizational structure. The literature on organizational theory contends that there are two types of organizational structure: mechanistic (inorganic) and organic ( Kyatuha et al., 2016). Related to the above, Rwothumio et al. (2021a) found out that “significant changes are taking place in organizations as a result of the great deviations in society.” According to him, the mechanical paradigm works best in settings with a high degree of inevitability, where technologies are frequently used, companies are sizable, and people are seen as just another resource. Vertical, practical, and bureaucratic structures are typical internal structures. The vertical structure and power disparities between superiors and subordinates indicate the organization’s application of rational analysis and its adherence to local norms. The organic model acknowledges the erratic, perhaps disordered, nature of the outside world. Because most technologies are not routine, the size is not as crucial. Organizations now place greater emphasis on collaboration, in-person communication, learning, and innovation. Traditional egalitarian values such as horizontal interactions, empowerment, equality, and consensus building have greater significance ( Keech, 2023). Organizational theorists have classified organizational structures as either mechanical or organic, while innovation researchers have identified distinctions between industrial and post-industrial operating styles. Modernization scholars suggest that as companies transition from an industrial to a post-industrial mode of operation, they require an organizational structure with several key attributes: minimal hierarchical layers to facilitate rapid responses, a high degree of horizontal integration to enhance knowledge transfer, decentralized decision making to address operational issues swiftly and effectively, and a high level of horizontal integration ( Mugizi & Nuwatuhaire, 2019).

The external environment of a company affects its organizational structure. According to research, businesses structured to manage complex, regularly shifting circumstances may not be as effective in predictable and stable markets ( Keech, 2023). The likelihood that a company’s organizational structure will have a centralized ladder and defined standards and processes increases with the level of security in the environment. High levels of conservational uncertainty may lead organizations to decentralize decision-making ( Mugizi & Nuwatuhaire 2019). In contrast, Keech (2023) asserted that a detailed analysis of organizational literature yielded a comprehensive list of structural characteristics. He noted that in their investigation of organizational determinants, researchers used specialized, functionally different, professional, formalized, centralized, administrative attitudes toward change, decision-making tenure, technical knowledge resources, level of administrative activity, external and internal communication, and vertical differentiation. Mugizi and Nuwatuhaire (2019) highlighted specialization, delegation, and mixing in discussing the role of context and structure in implementing logistical innovations, a list was provided that includes formalization, specialization, standardization, hierarchy of authority, complexity, centralization, professionalism, and staff ratios. Once more, it was stated that formalization, centralization, and participation helped explain the connections between environmental uncertainty and distribution channel bureaucracy.

In relation to the above, the four most frequently mentioned sub-dimensions of organizational structure are detailed below: formalization style, levels of hierarchy, degree of horizontal integration, and centralization of power ( Mugizi & Nuwatuhaire, 2019).

Related to the above is the formalization nature. The degree to which rules and processes are given to academic staff to prevent rather than promote innovative, independent work and learning defines formalization. According to the literature on organizational theory, there are two formalization levels: low formalization is associated with organic structures and high formalization is associated with mechanistic structures ( Fumasoli et al., 2020). According to research on innovation, flexible work regulations encourage invention, whereas a high level of formalization has the opposite effect ( Fumasoli et al., 2020). Bibi et al. (2020) stated that the hierarchy of organic organizations has few tiers. The assumption in the literature on innovation is that hierarchical levels lengthen the links between channels, making it more difficult to communicate between levels and reducing the flow of innovative ideas ( Fumasoli & Hladchenko, 2024). Integration at the horizontal level determines the quantity of horizontal integration by comparing functionally specialized subdivisions and academic staff to those who are integrated in their training, job, and skills (a high level of horizontal integration) ( Fumasoli & Hladchenko, 2024). Organizations often divide functional departments in line with the principle of division of labor so that tasks may be completed in order ( Davenport & Nohria, 1994). To respond to environmental changes and provide value to consumers, postmodern organizations organize their workforce into independent work teams, cross-functional teams, and task forces. To better understand the entire course and adapt to changing customer demands, staff members generally undergo cross-training ( Fumasoli & Hladchenko, 2024).

Similarly, decision making by locus refers to the proportion of choices made lower than higher in the organizational hierarchy ( Mugizi, Nuwatuhaire & Turyamureeba, 2019b). Organizations that follow the management control paradigm prioritize management rights, power locations, and status symbols. In organizations operating in such environments, decisions should be delegated to a level where academic staff can quickly adapt to changing conditions and deliver value to their customers ( Fumasoli & Hladchenko, 2024).

In addition, organizations are the most efficient and sensible social formations in society; thus, contemporary society relies on them. Organizations exist as social tools that coordinate human actions. The company can review its performance and modify accordingly while merging personnel, resources, and materials to be effective in accomplishing its goals ( Mugizi, Nuwatuhaire & Turyamureeba, 2019b). Related to the above ( Mugizi, Nuwatuhaire & Turyamureeba, 2019b), argued that structure refers to the relationships among the parts of a well-organized system. In organizational theory, social structure refers to the relationships among individuals, roles, and the organizational units to which they belong, such as departments and divisions. According to Weber, the key elements of an organizational structure are the hierarchy of authority, division of work, and rules and procedures. A detailed analysis of organizational structure and its components examines how these elements are interconnected and influence the overall structure of the organization. Weber argued that an organization’s structure determines its work distribution, reporting relationships, and formal coordination methods ( Mugizi, Nuwatuhaire & Turyamureeba, 2019b).

The organizational structure has three components: complexity, formalization, and centralization. Structural complexity refers to the extent of differentiation or division of work within an organization. A complicated organization requires more communication between departments, horizontally or vertically between levels. The greater the complexity of an organization, the greater the requirement for effective communication, coordination, and control ( Anwar, Mahmood, Yusliza, Ramayah, Faezah & Khalid, 2020). The impact of policies and procedures on organizational behavior is influenced by the level of formalization. There is a relationship between formalization and complexity, with high complexity often leading to low formalization owing to specialized expertise in highly complex organizations. Formalization tends to impede innovation and negatively affects communication within an organization because it involves numerous rules and procedures that dictate how tasks should be performed ( Mugizi & Nuwatuhaire, 2019).

Several scholars have demonstrated that the design of organizational structure directly influences institutional efficiency and staff motivation in higher education. For instance, hierarchical systems enhance control and accountability but often constrain innovation and participation ( Marisa & Oigo, 2018; Mugizi et al., 2019; Atwebembeire & Malunda, 2019). In the context of private universities in Uganda, structural rigidity has been linked to communication breakdowns and reduced staff morale, suggesting a need for more flexible arrangements that balance control with autonomy.

Mechanistic structures emphasize centralized control and formal rules, which often ensure efficiency but limit innovation. In contrast, organic structures represent a shift toward flexibility and collaboration, encouraging participatory decision-making and staff creativity ( Silaji et al., 2023; Rwothumio & Amwine, 2021a).

Studies in Uganda show that most private universities adopt hierarchical systems characterized by centralized authority and formal reporting lines. Building on these empirical insights, theoretical perspectives emphasize that effective structures must align with institutional goals to achieve sustainable performance outcomes ( Eze et al., 2024; Turyamureeba & Atwebembeire, 2020).

## Research gap and contribution

Despite a growing body of literature examining organizational structures and their influence on performance in higher education, most studies have focused on public universities or corporate organizations in developed countries ( Marisa & Oigo, 2018; Mugizi et al., 2019; Rwothumio & Amwine, 2021a). Existing research in Uganda has primarily explored administrative challenges, leadership practices, and governance dynamics ( Atwebembeire & Malunda, 2019; Nuwatuhaire & Turyamureeba, 2019), with limited emphasis on how the specific types of organizational structures hierarchical, functional, or matrix affect academic staff performance in private chartered universities. Moreover, prior studies have often treated organizational structure as a static administrative framework rather than a dynamic system influencing motivation, innovation, and accountability ( Silaji et al., 2024; Eze et al., 2024). This finding aligns with Rwothumio & Amwine (2021a), who noted that private universities in Uganda employ hierarchical structures.

Therefore, a significant empirical and contextual gap exists regarding how structural arrangements shape academic staff performance in Uganda’s rapidly expanding private university sector, where institutional governance is still evolving under limited resources and regulatory oversight. This study addresses that gap by examining the types of organizational structures used in private chartered universities in Western Uganda and analyzing their impact on academic staff performance. It contributes to both organizational theory and higher education management practice by extending Henri Fayol’s Administrative Management Theory and Vroom’s Expectancy Theory to a developing-country context. Practically, the study offers evidence-based insights to guide university leaders and policymakers toward adopting adaptive and participatory structures that enhance staff performance, motivation, and institutional effectiveness.

## Theoretical framework

This study draws upon Contingency Theory, which posits that the effectiveness of an organizational structure is contingent on how well it aligns with the institution’s environment and goals ( Lawrence & Lorsch, 1967). In the context of private universities in Uganda, this theory helps explain why certain structures are more effective than others, based on institutional size, faculty composition, and educational goals. Additionally, Mintzberg’s organizational configuration theory provides a framework for understanding how different structures—hierarchical, functional, and matrix—affect communication, decision-making, and staff performance ( Mintzberg, 1979).

## Methodology

A mixed-methods approach was employed to collect both the quantitative and qualitative data. The quantitative component involved a survey administered to 186 academic staff members from two privately chartered universities in Western Uganda. The survey focused on identifying the types of organizational structures used within the institutions and how these structures influenced staff performance. The qualitative component involved in-depth interviews with ten deans from various faculties. The interviews aimed to provide insights into how university management perceives the impact of organizational structure on academic staff performance, and the benefits and challenges of the adopted structures.

All participants involved in this study provided informed consent prior to their participation. The consent process was conducted in accordance with the ethical standards of the Kampala International University Research Ethics Committee (approval number: **KIU-2024-292**) and the Uganda National Council for Science and Technology (approval number: **SS3145ES**).

Participants were thoroughly informed about the study’s purpose, procedures, potential risks and benefits, confidentiality measures, and their rights, including the right to withdraw from the study at any time without any consequences. Consent was obtained in writing, ensuring that participants had adequate time to consider their involvement and had the opportunity to ask questions. This consent process adhered to international ethical principles, including those outlined in the Declaration of Helsinki and the Belmont Report, ensuring respect for persons, beneficence, and justice throughout the research.

Inferential statistical techniques were employed because the study aimed to examine relationships among variables rather than to describe distributions only. Although the data were collected using Likert-scale items, composite indices were computed from multiple items measuring each construct. According to methodological literature, aggregated Likert-scale measures approximate interval-level data and can be analysed using parametric statistics when normality assumptions are reasonably satisfied. Preliminary diagnostics indicated acceptable reliability and distribution characteristics; therefore, correlation and regression analyses were appropriate for examining the strength and direction of relationships between organisational structure, performance monitoring, and academic staff performance.

More advanced multivariate techniques such as structural equation modelling and factor analysis were not adopted because the study did not aim to develop or validate a new measurement model but rather to test associations among already established constructs. Additionally, the study objectives focused on predictive relationships rather than latent construct modelling. Non-parametric methods were also considered less suitable because they limit estimation of predictive magnitude and explanatory variance, which were central to the study.

### Validity and reliability

The questionnaire was reviewed by three experts in educational management and institutional governance to ensure content validity and construct relevance. A pilot test involving 50 academic staff from a non-sampled university was conducted to test clarity, reliability, and internal consistency.

**Table 1.** Content Validity Index (CVI) of the Research Instrument.

Item category	Number of items (N)	Items rated as relevant (n)	CVI = n/N	Interpretation
**Organizational Structure**	25	23	0.92	Excellent validity
**Academic Staff Performance**	25	23	0.92	Excellent validity
**Overall Instrument**	**75**	**68**	**0.91**	**Strong content validity**

### Interpretation paragraph

The validity of the research instrument was assessed using the Content Validity Index (CVI) approach, as proposed by Lynn (1986). The instrument was reviewed by a panel of five experts in higher education management and research methodology. Each item was rated for relevance on a 4-point scale ranging from not relevant (1) to highly relevant (4). The CVI was computed using the formula:

CVI=n/N

where n is the number of items rated as relevant (scores of 3 or 4), and N is the total number of items assessed. Out of 75 items, 68 were rated as relevant, yielding a CVI of 0.91. According to Polit and Beck (2006), a CVI above 0.80 indicates strong content validity. Therefore, the overall CVI of 0.91 confirms that the instrument demonstrated excellent content validity, ensuring that the items adequately captured the constructs of organizational structure, performance monitoring, and academic staff performance.

### Interpretation paragraph

The internal consistency reliability of the research instrument was tested using Cronbach’s Alpha (α) coefficient for each construct. According to Nunnally (1978), a reliability coefficient of 0.70 or higher indicates acceptable internal consistency, while values above 0.80 are considered good to excellent.

As presented in Table 2, all constructs demonstrated high reliability, with Cronbach’s alpha values ranging between 0.87 and 0.91. Specifically, the academic staff performance scale had the highest alpha (α = 0.91), indicating excellent internal consistency among its items. The organizational structure (α = 0.89) and performance monitoring (α = 0.87) dimensions also displayed strong reliability, confirming that the measurement items were consistently understood and interpreted by respondents.

**Table 2.** Showing Reliability Results of the Research Questionnaires “Report reliability coefficients (Cronbach’s alpha or composite reliability) for each construct.”

Construct/Variable	Number of Items	Cronbach’s Alpha (α)	Interpretation
**Organizational Structure**	25	0.89	High reliability
**Academic Staff Performance**	25	0.91	Excellent reliability
**Overall Instrument**	**75**	**0.89**	**Highly reliable**

The overall instrument achieved a composite Cronbach’s alpha of 0.89, suggesting that the instrument is highly reliable and suitable for further statistical analysis. This reliability outcome aligns with recommendations by George and Mallery (2019), who classify alpha values above 0.80 as evidence of good reliability.

The study was conducted in private chartered universities located in Western Uganda, including institutions accredited by the National Council for Higher Education (NCHE) such as Bishop Stuart University and Kampala International University–Western Campus.

The target population comprised 386 academic staff members and 10 academic deans, representing faculties of education, business, science, and humanities. Academic staff were included to capture performance-related experiences, while deans were selected for their managerial insights into institutional structures and performance systems.

### Sampling procedure and sample size determination

A stratified random sampling technique was used to ensure representation across different faculties and academic ranks (professor, senior lecturer, lecturer, and assistant lecturer). Each university was treated as a stratum, and respondents were proportionately selected based on the size of the faculty population.

The sample size was determined using Krejcie and Morgan’s (1970) sample size determination table for finite populations. Out of 420 academic staff, a total of 386 respondents were selected for the quantitative phase.

For the qualitative phase, 10 deans were purposively selected based on their administrative roles, years of experience, and willingness to participate. This number was sufficient to achieve data saturation, as recommended by Guest, Namey, and Chen (2020), ensuring that no new themes emerged beyond the tenth interview.

### Inclusion criteria

The inclusion criteria for this study involved Deans, Head of departments, and Lecturers who are mentally stable and had consented to participate in this study. Therefore, these academic staff members were directly involved in the core functions of teaching, research, and academic administration, which are crucial to the performance of universities. Hence, the Deans and Heads of Departments play a significant role in decision-making, strategy implementation, and department-level leadership, which directly affects academic staff performance. While the Lecturers are frontline educators and researchers, whose job performance is essential for the success of the institution. So, focusing on these groups allows the study to assess performance-related variables among those most engaged in academic activities. Another inclusion criteria considered was mentally Stable Participants: Ensuring that participants are mentally stable is necessary to obtain valid, reliable, and ethical data. Mental stability ensures that respondents can accurately and meaningfully engage with the research process, answer questions objectively, and make informed decisions about their participation. This is critical for the credibility and ethical integrity of the study. In addition, Participants Who had Consented: Consent is a fundamental ethical requirement in any research involving human participants. Ensuring that only those who consent participate protects the rights and autonomy of individuals. It also complies with ethical standards in research, which emphasize voluntary participation, informed consent, and the right to withdraw at any time.

Furthermore, Private Chartered universities which met specific government standards for higher education, representing a formalized and structured environment for academic activities. By focusing on these institutions allowed the study to assess performance within officially recognized and accredited universities, ensuring that findings are relevant and applicable to private higher education in the region. Hence limiting the study to Western Uganda which narrowed the geographical scope, making the research more manageable and context-specific while maintaining a consistent level of institutional development and governance structures.

And finally, the inclusion of research assistants also enhanced efficiency, broadened coverage, and reduced the likelihood of researcher bias during fieldwork.

### Exclusion criteria

This study excluded other academic staffs like Top management members, students, supporting staff members like Finance department, security guards, whoever shall not consent to participate.

The study excluded individuals and institutions whose participation would not directly contribute to understanding academic staff performance in chartered private universities. Specifically, top management members, such as Vice Chancellors, University Secretaries, and Registrars, were excluded because their roles focus on high-level strategic decisions rather than departmental academic operations. Students were excluded as they are recipients of educational services rather than providers, and supporting staff, including those in the Finance Department or security, were excluded because their roles are not directly linked to academic performance. Non-consenting individuals were also excluded to uphold ethical standards of voluntary participation. Finally, non-chartered private universities were excluded, as they may not meet regulatory standards, and including them could introduce variability in institutional quality and governance structures. This focus ensured consistency in the academic environment and maintains the study’s emphasis on investigating types of organizational structure, performance monitoring factors that influence academic staff performance.

### Data collection instruments

*Quantitative data*

The quantitative instrument was a structured self-administered questionnaire divided into three sections:

1. Section A: Demographic information (age, gender, education, rank, and experience).
2. Section B: Items on Organizational Structure Dimensions (Hierarchy & Chain of Command, Departmentalization, Centralization & Decentralization, and Formalization), adapted from Daft (2016) and modified for the university context.
3. Section C: Items on Academic Staff Performance (Teaching Effectiveness, Research & Publication, Student Mentoring & Support, Professional Development, and Community Service), adapted from Mugizi et al. (2019) and Turyamureeba & Atwebembeire (2020).

All items were measured on a five-point Likert scale ranging from 1 (Strongly Disagree) to 5 (Strongly Agree).

*Qualitative data*

Qualitative data were collected using a semi-structured interview guide designed to elicit detailed narratives on organizational structure and its perceived effect on academic performance. The guide consisted of five main questions focusing on:

1. Hierarchical relationships and decision-making processes;
2. Communication flow and collaboration;
3. Performance evaluation and accountability systems;
4. Resource distribution and institutional support; and
5. Perceived influence of structure on staff motivation and productivity.

Each interview lasted approximately 45–60 minutes, conducted face-to-face in English. With participants’ consent, the interviews were audio-recorded and later transcribed verbatim to ensure accuracy. Field notes were taken to capture non-verbal cues and contextual insights.

### Data analysis procedures

*Quantitative analysis*

Quantitative data were analyzed using the Statistical Package for Social Sciences (SPSS) Version 26. Descriptive statistics (frequencies, percentages, means, and standard deviations) summarized respondents’ views. Inferential statistics were used as follows:

• Pearson’s Linear Correlation Coefficient (PLCC) to determine the relationship between organizational structure dimensions and academic staff performance.
• Analysis of Variance (ANOVA) to identify significant differences across structure dimensions.
• Multiple Regression Analysis to examine the predictive influence of organizational structure dimensions on performance outcomes.

Regression assumptions normality, linearity, homoscedasticity, and multicollinearity were tested and satisfied. The coefficient of determination (R ^2^), standardized beta (β), F-statistics, and p-values were reported. Statistical significance was set at p < 0.05, with confidence intervals computed at 95%.

*Qualitative analysis*

Qualitative data were analyzed using Braun and Clarke’s (2006) six-step thematic analysis framework, which involved:

1. Familiarization with the data,
2. Generating initial codes,
3. Searching for themes,
4. Reviewing themes,
5. Defining and naming themes, and
6. Producing the final narrative report.

NVivo software (version 12) facilitated coding and data organization. Themes that emerged included hierarchical relationships, centralized decision-making, communication and trust, institutional support, and autonomy in performance.

To ensure credibility and trustworthiness, the researcher employed:

• Member checking, where summaries of interpretations were verified by participants;
• Peer debriefing with two research colleagues; and
• Data triangulation, by integrating quantitative and qualitative results.

### Ethical considerations

*Ethical considerations statement*

This study was conducted in accordance with the ethical standards set forth by the American Physical Society (APS) and adhered to all relevant institutional and national guidelines. Ethical approval was obtained from the Kampala Interna- tional University Research Ethics Committee (KIU-REC) under approval number KIU-2024-292, and from the Uganda National Council for Science and Technology (UNCST) under national approval number SS3145ES. uncst.go.ug Permission to conduct the study was granted by participating universities. Respondents were informed about the purpose of the study, confidentiality procedures, and their right to withdraw at any time. Informed consent was obtained before data collection. To protect anonymity, pseudonyms (e.g., DU PT1–DU PT10) were used in reporting qualitative quotes.

### Summary

In summary, this methodology section ensures replicability, transparency, and rigor through a clearly defined sampling procedure, validated instruments, triangulated analysis, and ethical integrity. The mixed-methods design provides both breadth and depth in understanding how organizational structures influence academic staff performance, aligning with best practices in educational management research.

## Findings

### The descriptive statistics of types of organisational structure in private universities

This section deals with quantitative data analysis, which is evidence-based or based on numbers ( Kotronoulas et al., 2023), and involves numerical and graphical techniques that briefly summarize a dataset, making it clear and understandable ( Cooksey, & Cooksey, 2020). Descriptive statistics for summarizing data. *Illustrating statistical procedures: Finding meaning in quantitative data*, 61-139.). Each variable was examined independently to determine the range of values in a dataset and assess how evenly they were distributed. To explore the relationship between organizational structure, performance monitoring, and Academic Staff performance in private Universities in Western Uganda, a descriptive analysis of data on the independent variables, which are the types of organizational structure in private universities and types of performance monitoring used in private universities **.** types of organizational structures in private universities were first conducted and are presented in this section. This section presents items on hierarchy and chain of command, departmentalization, centralization, decentralization, and formalization, measured using a five-point Likert scale ranging from 1 to 5:1 = Strongly Disagree (SD), 2 = disagree (D), 3 = neutral (U), 4 = agree (A), and 5 = Strongly Agree (SA).

**Hierarchy and chain of command**

Using five measures, this was the first area of organizational structure in Private Universities to be examined. The results are shown in Table 3.

**Table 3.** Frequencies, percentages, and means for hierarchy and chain of command.

S/No	Hierarchy and chain of command	F (%)	SD	D	N	A	SA	Mean
B1.1	The university has a clear hierarchical structure.	F (%)	4 (1.0)	25 (6.5)	43 (11.1)	174 (45.1)	140 (36.3)	4.09
B1.2	There is a well-defined chain of command.	F (%)	0 (0)	0 (0)	3 (3.3)	35 (38.9)	52 (57.8)	3.83
B1.3	Decision-making authority is appropriately distributed across different levels.	F (%)	0 (0)	0 (0)	1 (1.1)	39 (43.3)	50 (55.6)	3.91
B1.4	The roles and responsibilities at each level are clearly defined.	F (%)	0 (0)	0 (0)	11 (12.2)	41 (45.6)	38 (42.2)	3.91
B1.5	The organizational structure supports effective communication.	F (%)	1 (1.1)	0 (0)	3 (3.3)	39 (43.3)	47 (52.2)	3.90
	**Grand mean**							3.93
	**Remarks: good**							

The findings in Table 3 concerning whether the university has a clear hierarchical structure, about 81.4% (Agree + Strongly Agree) support this statement, with minimal disagreement (only 7.5 **%** disagree or strongly disagree). The mean was 4.09, the highest-rated item, indicating a strong consensus that the university has a clear hierarchical structure. The result of a well-defined chain of commands showed that 96.7% (Agree + Strongly Agree) agreed, and no respondents strongly disagreed or disagreed. The mean of 3.83, means the item is also rated positively. This finding reinforces the existence of a robust command structure.

Furthermore, it is important to know whether decision-making authority is appropriately distributed, and the results showed that the majority agreed (98.9% Agree + Strongly Agree), suggesting that decision-making is well distributed. Only 1.1% of patients remained neutral. This was supported by the high mean of 3.91, close to code 4, which on the scale used corresponded with agreement. Moreover, in the data on whether roles and responsibilities are clearly defined, the statement is perceived positively by 87.8% of respondents (Agree + Strongly Agree), showing clarity in organizational roles, while 12.2% remain neutral, with a mean of 3.91.

Finally, the organizational structure supporting communication revealed that 95.5% of the respondents agreed that the structure supports effective communication, underscoring the functional alignment of the hierarchy, while only 1.1% disagreed, with a mean of 3.90. A grand mean of 3.93 indicates an overall good hierarchy and chain of command among respondents. The absence of significant negative responses (Strongly Disagree or Disagree) for most items shows an organizational consensus on the effectiveness of these aspects. The data reflect strong organizational confidence in the hierarchical structure and chain of command, suggesting effective governance, role clarity, and communication.

**Departmentalization**

This measure is the second aspect of the types of organizational structure in private universities studied, using five items. The results are also presented in Table 4 below.

**Table 4.** A table showing departmentalization.

S/No	Departmentalization	F (%)	SD	D	U	A	SA	Mean	STD
B1.6	The university is divided into well-defined departments or faculties.	F (%)	4(1)	35 (9.1)	56 (14.5)	184 (47.7)	107 (27.7)	3.91	0.95
B1.7	Each department has a specific focus and objectives.	F (%)	7(1.8)	50 (13.0)	70 (18.1)	175 (45.3)	84 (21.8)	3.72	1.01
B1.8	There is effective coordination between different departments.	F (%)	12 (3.1)	28 (7.3)	51 (13.2)	204 (52.8)	91 (23.6)	3.86	0.98
B1.9	Departments have the autonomy to manage their operations.	F (%)	0(0)	28 (7.2)	68 (17.6)	179(46.4)	111 (28.8)	3.96	0.90
B1.10	Interdepartmental collaboration is encouraged and facilitated.	F (%)	10 (2.6)	31 (8.0)	58 (15.0)	213 (55.2)	74 (19.2)	3.80	0.95
	**Grand mean**							3.85	

The data in Table 4 concerning departmentalization, the result on whether the university is divided into well-defined departments or faculties, shows that 75.4% agree and strongly agree. In comparison, 14.5% were neutral and only 10.1% disagreed. A mean score of 3.91 indicates high agreement that the university was structured into clearly defined departments. Whether each department has a specific focus and objective reveals that 67.1% agree or strongly agree, 18.1% are neutral, and 13.1% disagree. A mean of 3.72, reflects the high agreement that departments have distinct focuses and objectives. In addition, regarding departmentalization, data on whether there is effective coordination between different departments shows that 76.4% agree or strongly agree, although 13.2% are neutral and 10.4% disagree. A mean of 3.86 suggests high agreement that interdepartmental coordination is effective.

Departments have autonomy to manage their operations, and 75.2% support the statement, with no respondents strongly disagreeing. A mean of 3.96 reflects the highest level of agreement in the table. Finally, regarding the question of whether interdepartmental collaboration is encouraged and facilitated, 74.4% agree or strongly agree, although 15.0% remain neutral, while 10.6 disagree. A mean of 3.80 indicates high agreement that collaboration between departments is supported. Overall Interpretation Grand Mean = 3.85, which reflects an overall high agreement with the effectiveness of departmentalization within the university. The responses indicated that most participants perceived the university’s departmental structure and practices positively.

**Centralization and decentralization**

This is the third aspect of organizational structure in private universities studied, using five items. The results are presented in Table 5.

**Table 5.** A table showing Centralization and Decentralization.

S/No	Centralization and Decentralization	F (%)	SD	D	U	A	SA	Mean	STD
B1.11	Decision-making is centralized at the top levels of the university.	F (%)	1 (0.3)	13 (3.4)	41 (10.6)	170 (44.0)	161 (41.7)	4.24	0.79
B1.12	Departments have the authority to make decisions within their scope.	F (%)	1 (0.3)	10 (2.6)	61 (15.8)	230 (59.6)	84 (21.8)	4.00	0.71
B1.13	There is a balance between central control and departmental autonomy.	F (%)	24 (6.2)	10 (2.6)	66 (17.1)	212 (54.9)	74 (19.2)	3.78	0.99
B1.14	The university's structure supports both central oversight and local innovation.	F (%)	17 (4.4)	3 (0.8)	48 (12.4)	221 (57.3)	97 (25.1)	3.98	0.90
B1.15	Communication flows effectively between the central administration and departments.	F (%)	3 (0.8)	15 (3.9)	47 (12.2)	235 (60.9)	86 (22.3)	4.00	0.76
	**Grand mean**							4.00	

### Analysis and interpretation of the Table 5

This table presents the results of academic staff perceptions of centralization and decentralization in a university setting, focusing on decision-making, departmental autonomy, and communication dynamics. Each statement was rated on a 5-point Likert scale, with the following findings: the results on centralized decision-making showed that a significant majority agreed or strongly agreed that decision-making is centralized at the top levels, with 44% agreeing and 41.7% strongly agreeing (85.7% combined). Very few disagreements (3.4%) or remained neutral (10.6%) indicated a strong consensus. The mean is 4.24, corresponding to the Likert scale 5 = Strongly Agree The average response is close to “Strongly Agree.” This suggests a strong consensus that decision making is centralized at the top level of the university. The centralization of decision-making is a prominent feature of the organizational structure. The standard deviation is 0.79, showing moderate variability in responses, while most respondents agree on centralized decision making, and there are some outliers or differing opinions. The majority shared similar views. This suggests a strong perception of top-level control in universities’ decision-making processes.

The data on departmental authority showed that a combined 81.4% agree (59.6%) and strongly agree (21.8%) that departments have the authority to make decisions within their scope. A small proportion disagreed (2.6%), while (15.8%). remain neutral. This indicates that while departments are perceived to have decision-making autonomy, the high meanof 4.00 suggests broad agreement with this sentiment. A standard deviation of 0.71 indicates that responses are clustered close to the mean. This suggests that most respondents have similar opinions regarding departmental decision-making authority. There was strong consensus or agreement among respondents regarding this item.

The results on whether there is a balance between central control and autonomy showed that 54.9% agreed and 19.2% strongly agreed (74.1% combined) that there is a balance between central control and departmental autonomy, with 6.2% disagreeing and 17.1% neutral, suggesting some uncertainty about this balance. with a mean of 3.78 and a standard deviation of 0.99. Although there was moderate agreement, a higher standard deviation (0.99) reflected diverse opinions or less consensus among respondents. The results of the support for central oversight and innovation revealed that 82.4% (57.3% agreed, 25.1% strongly agreed) perceived the university’s structure as supportive of both central oversight and local innovation. Only 5.2% disagreed, with 12.4% neutral. Mean: 3.98 and Standard Deviation: 0.90 This reflects a positive view that the structure balances oversight with opportunities for innovation, albeit with some variability in responses.

Finally, regarding effective communication, an overwhelming 83.2% agreed (60.9%) or strongly agreed (22.3%) that communication flows effectively between central administration and departments. Very few respondents either disagreed (3.9%) or remained neutral (12.2%). With a Mean of 4.00 and a Standard Deviation of 0.76, communication is widely seen as effective, contributing positively to organizational functioning. Overall Perception (Grand Mean = 4.00), an overall grand mean of 4.00, indicates strong agreement with statements regarding centralization and decentralization. The low standard deviations across items suggest consistency in responses, except for B1.13, where variability was slightly higher.

The results suggest a predominantly positive perception of the university’s structure in balancing centralization and decentralization. Decision-making is centralized, with adequate departmental autonomy and effective communication supporting organizational cohesion. However, there is room for improvement in achieving a perceived balance between central control and departmental autonomy, as indicated by B1.13.

### Analysis and interpretation of formalization Table 6

**Table 6.** A table showing the formalization.

S/No	Formalization	F (%)	SD	D	U	A	SA	Mean	STD
B1.16	The university has well-documented policies and procedures.	F (%)	28 (7.3)	12 (3.1)	26 (6.7)	189 (49.0)	131 (33.9)	3.99	1.09
B1.17	There is a high degree of formalization in administrative processes.	F (%)	14 (3.6)	19 (4.9)	42 (10.9)	195 (50.5)	116 (30.1)	3.98	0.97
B1.18	Staff members are well-informed about formal policies and procedures.	F (%)	15 (3.9)	28 (7.3)	52 (13.5)	187 (48.4)	104 (26.9)	3.87	1.02
B1.19	The formalization supports consistency and accountability.	F (%)	16 (4.1)	27 (7.0)	58 (15.0)	213 (55.2)	72 (18.7)	3.77	0,97
B1.20	The university's policies are regularly reviewed and updated.	F (%)	19 (4.9)	27 (7.0)	48 (12.4)	193 (50.0)	99 (25.6)	3.84	1.04
	**Grand mean**							3.89	

The mean values for the items in the “Formalization” category range from **3.77** to **3.99**, indicating varying levels of agreement with the statements. On a Likert scale (1 = Strongly Disagree, 5 = Strongly Agree), these means indicate that respondents generally agree with the statements, but their perceptions vary.

To check if there are well-documented policies and procedures (Mean = 3.99, STD = 1.09), the average response is close to “Agree,” indicating that respondents perceive the university to have well-documented policies and procedures. Standard deviation (STD) had relatively high variability (1.09), suggesting differing opinions among respondents, with some strongly disagreeing and others strongly agreeing. Furthermore, formalization in administrative processes indicates that mean = 3.98, and STD = 0.97), meaning that respondents generally agree that administrative processes are highly formalized. Standard Deviation: A Moderate variability (0.97) indicated slight differences in perception.

In addition, awareness of formal policies and procedures Mean = 3.87, and STD = 1.02, meaning that respondents agreed that staff members were informed about policies and procedures, though slightly less strongly than the above. The standard deviation of moderate variability (1.02) showed some inconsistency in agreement levels. Formalization supports consistency, and accountability results show that mean = 3.77, and STD = 0.97. This is the lowest mean among the items, suggesting that respondents perceived formalization’s support for consistency and accountability slightly less positively. The standard Deviation indicated moderate variability (0.97), reflecting a consistent pattern of perceptions. Policies are regularly reviewed and updated, showing a mean of 3.84, and an STD of 1.04. Mean Explanation: Respondents agree that the university regularly reviews and updates policies, though not as strongly as they agree with B1.16 or B1.17. The standard Deviation shows variability (1.04), indicating some divergence.

Grand Mean = ~3.89: On average, respondents agreed with statements about the university’s formalization practices. However, none of the items achieve a “Strongly Agree” consensus (mean ≥4.0), suggesting there is room for improvement. The findings suggest that the university has a moderately strong formalization structure, with policies and processes generally well documented and perceived positively. However, there is little consensus on whether formalization effectively supports consistency and accountability, or ensures regular policy updates. Efforts to address these issues could enhance the overall perception of formalization within universities ( Table 7).

**Table 7.** Ranking table of the types of organizational structures.

Rank	Types of organizational structure	Mean score	Agreement (%)
1	Centralization and Decentralization	4.00	80%
2	Hierarchy	3.93	75%
3	Formalization	3.89	65%
4	Departmentalization	3.85	70%

The top organizational structure is “Centralization and Decentralization”, which is the most prominent with a mean score of 4.00 and an 80% agreement rate. The least type of organizational structure is “Departmentalization” which ranks lowest with a mean score of 3.85, despite a 70% agreement rate. The first objective of this study is to determine the types of organizational structures used in private universities in western Uganda, showing that Centralization and Decentralization are the types of organizational structures used in private universities in western Uganda.

Quantitative Results: The survey responses revealed the following distribution of organizational structures.

Hierarchical Structure: Sixty% of respondents reported that their university employed a hierarchical structure characterized by a clear chain of command and centralized decision-making.

Functional Structure: Thirty% of the respondents indicated that their universities used a functional structure, where departments operate with a high degree of specialization (e.g., separate departments for teaching, research, and administration).

Matrix Structure: Ten% of the respondents reported a matrix structure that combines elements of both hierarchical and functional models, allowing for flexibility in staffing and task management.

These structures are reflected in the respondents’ perceptions of their roles and performance:

In hierarchical institutions, 70% of the staff members indicated clear expectations and well-defined roles.

In functional institutions, 65% felt that they could specialize and focus on specific academic tasks, but 40% mentioned occasional communication issues.

In matrix-based institutions, 80% of the staff reported improved collaboration and innovation, but 50% expressed concerns about role ambiguity.

Qualitative Results: Deans highlighted the strengths and weaknesses of different structures:

“In a hierarchical structure, we have clear control and oversight, but sometimes it limits faculty autonomy, especially in research.” (Dean 1, DU PT1-32)

“The functional structure allows for specialized academic work, but it can lead to siloed departments, which hampers interdisciplinary collaboration.” (Dean 3, DU PT3-41)

“The matrix structure works well for interdisciplinary research, but it can be confusing for staff when it comes to reporting lines.” (Dean 6, DU PT6-50)

These insights reflect the varying impacts of different structures on academic staff performance, highlighting the trade-off between clarity and flexibility.

## Discussion

The findings reveal that most private universities in Western Uganda have adopted hierarchical or functional structures, with a small number experimenting with matrix structures. Hierarchical structures are associated with clear authority lines and decision-making processes that can enhance administrative efficiency. However, these structures may limit academic freedom and innovation, particularly in the research areas.

Functional structures offer greater specialization, which is beneficial for academic staff focusing on specific tasks. However, these structures can create silos and hinder cross-departmental collaboration, which is essential for academic growth. Although less common, the matrix structure provides a flexible approach that supports collaboration among departments. However, the complexity of dual reporting relationships can lead to confusion and role ambiguity, particularly in larger institutions.

The integration of quantitative and qualitative findings indicates that the prevailing organizational structures in these universities are predominantly functional and hierarchical. While such models ensure clear lines of authority and decision-making efficiency, they often hinder innovation and diminish the motivation of academic staff. This is corroborated by the significant correlation (r = 0.53) and R ^2^ = 0.284, suggesting that organizational structure accounts for a significant portion of the staff performance variance. The findings resonate with Yusoff and Isa’s (2021) assertion that rigid hierarchical structures may inhibit collaborative decision-making, thereby reducing academic productivity. Similarly, Berkowitz (2023) notes that governance models that fail to incorporate academic voices often lead to disengagement and turnover, a pattern also observed in some Ugandan private universities.

These findings are consistent with Rwothumio et al. (2021a) who noted that private universities in Uganda adopt hierarchical structures. Which also agrees with Rwothumio et al. (2021b) who found that monitoring systems improve academic accountability.”

Recent studies support these findings. For instance, Turyamureeba et al. (2023) observed that rigid hierarchical structures in Ugandan private universities limit staff autonomy and innovation, thus adversely affecting performance. Similarly, Mugizi et al. (2019b) found that, while formalization within organizational structures can enhance employee commitment, excessive centralization and complexity may impede it.

According to Mugizi et al. (2019d, 2019e, 2019f), private universities in Uganda often adopt mechanistic structures, consistent performance monitoring enhances accountability, and staff motivation mediates the link between monitoring and performance. 

Birungi et al. (2024) highlighted that effective performance management practices, including participatory decision-making and decentralized structures, positively influence academic staff performance in private universities.

These findings resonate with Adyanga, Sekiwu, and Ankunda (2022), who highlighted the need to integrate quality processes into university programs for national development.

Similarly, Agyemang and Ofei (2013) established that employee engagement and organizational commitment significantly enhance performance in both public and private institutions.

Also, these finding aligns with Agyemang and Broadbent (2015), who demonstrated that management control systems influence research performance evaluation in UK universities.

Collegial Structures ( Posselt et al., 2020):

Agreement: This study recommends participatory structures to improve academic performance. Posselt et al. (2020) found that collegial models increased faculty satisfaction and collaborative outputs, aligning with this qualitative data that calls for “flatter structures where innovation is encouraged” (Dean 8).

Thematic Overlap: Shared governance and decentralization boost engagement and performance, reinforcing these recommendations.

Divisional Structures ( Koigi et al., 2018):

Agreement: ( Koigi et al., 2018) reported improved staff performance (20%) and efficiency (30%) in divisional structures. This study supports the idea that rigid functional structures hinder autonomy and calls for more flexible alternatives such as decentralized or hybrid systems. This is in agreement with Ahmed and Gohar (2019), who reported that structured performance monitoring enhances academic staff productivity in private universities in Pakistan.

Methodological Consistency: ( Koigi et al., 2018) used a quantitative approach to strengthen comparative reliability.

Matrix Structures ( Sakthivel & Raju, 2020):

Agreement: Their findings that matrix structures foster innovation and performance (despite challenges) align with the identification of hybrid models in 25.3% of the cases. This suggests an emerging shift toward adaptable, cross-functional systems in higher education, consistent with the call for structural rethinking. This finding is consistent with Al Rashdi (2020), who found that performance-related pay positively affects employee performance in both public and private sectors.

Flat Structures ( Ndirangu & Udoto, 2021):

Agreement: Their finding that flat structures improved staff engagement (28%) and performance (17%) supports this dean’s call for less hierarchical systems. This qualitative data reflects the same sentiment, e.g., “we need flatter structures …”

This supports Abdulrahaman (2020), who observed that rigid hierarchical structures constrain innovation and reduce academic staff motivation in Nigerian universities.

**In Contrasts;**

Bureaucratic Structures ( Dedahanov et al., 2017):

Agreement in Critique: Although there is a contrast in structure, the negative impacts of bureaucracy on morale and decision-making mirror these findings. This study’s quote—“we are stuck in bureaucratic practices that demotivate staff” echoes this directly.

However, this study does not explicitly use the term “bureaucratic,” but its characteristics (rigid, top-down) are clearly present.

Leadership Style and Job Satisfaction ( Jameel & Ahmad, 2020):

Partial Contrast: This study focuses on organizational structure, while Jameel and Ahmad (2020) emphasize leadership styles and job satisfaction. However, their finding that transformational leadership improves performance complements this recommendation for structural reforms to enhance motivation.

Potential integration: The mediating variable (job satisfaction) could enrich this study’s theoretical framework, especially in relation to Vroom’s Expectancy Theory.

Systematic Review on Structure & Performance ( Siddiqui, 2022):

Mixed Agreement: While this study found a significant relationship between structure and academic staff performance (R ^2^ = 0.284), Siddiqui’s meta-analysis suggests inconsistent effects across sectors and structures. This adds nuances and supports the need for further research. These results support Rwothumio & Amwine (2021b), who found that performance monitoring enhances accountability and efficiency.

Broader implications: Siddiqui’s review justifies this suggestion for future studies on agility and innovation in HEIs.

How These Studies Support or Enrich This Work

This study stands on solid ground and is well-aligned with the current empirical literature.

Most of the reviewed studies (Posselt et al., Koigi et al., Sakthivel & Raju, Ndirangu & Udoto) directly validated the claim that participatory, decentralized, or hybrid structures foster better academic outcomes.

The conclusion that rigid hierarchical structures constrain performance is echoed in both quantitative metrics and qualitative feedback across studies.

Incorporating leadership style ( Jameel & Ahmad, 2020) and job satisfaction as mediators could further strengthen this model.

Siddiqui’s review supports the call for contextual studies specific to academic institutions in Uganda.

The predominance of hierarchical and formalized systems in these institutions reflects the administrative legacy of traditional management theories such as Fayol’s (1916), which advocate for clear authority lines, division of labor, and centralized control. These features promote consistency, supervision, and accountability attributes essential in maintaining academic standards. However, excessive rigidity may hinder staff motivation and adaptability, as highlighted by Silaji et al. (2025), who found that mechanistic structures often constrain innovation in higher education.

The integration of Vroom’s Expectancy Theory (1964) helps to interpret these findings further. According to Vroom, motivation depends on the expectation that effort will lead to desired outcomes. In universities where decisions are centralized and communication is top-down, staff may perceive limited control over outcomes, reducing expectancy and thus lowering motivation. This was echoed by Dean 9, who stated that “feedback sometimes comes across as fault-finding” (DU PT9-304), reflecting reduced trust in hierarchical feedback mechanisms.

Similarly, Eze et al. (2024) and Latif et al. (2024) note that participatory structures those allowing greater faculty input in governance enhance staff engagement and performance. These findings imply that while private chartered universities benefit from structured management systems, there is a need to introduce organic elements such as participative decision-making, open communication, and decentralization of authority to improve staff morale and innovation.

Furthermore, Rwothumio and Amwine (2021a) emphasize that academic institutions thrive under hybrid structures that balance bureaucracy with flexibility. This balance ensures that while accountability and supervision are maintained, staff still enjoy professional freedom and opportunities for creativity. Such a balance was partially reflected in Dean 4’s observation of a “supportive” yet formal relationship, demonstrating that structured systems can still foster collaboration when guided by mutual respect and developmental intent.

In summary, the findings confirm that organizational structure significantly shapes academic staff performance. Hierarchical and centralized systems enhance coordination but limit autonomy, suggesting that universities should reconfigure their structures toward participatory and adaptive models. By doing so, they can align institutional efficiency with academic motivation an approach supported by Fayol’s managerial coordination principles and Vroom’s motivational theory.

## Conclusion for objective one

The study concludes that private chartered universities in Western Uganda predominantly use mechanistic structures notably hierarchical and functional arrangements with limited decentralization. While these structures promote discipline and accountability, they can hinder creativity and staff engagement if not balanced with participatory practices. To enhance academic performance, university leaders should adopt hybrid organizational structures that integrate hierarchical clarity with collaborative decision-making, transparency, and staff empowerment. Such adaptive structures can foster innovation, strengthen communication, and sustain institutional effectiveness, aligning with the evolving demands of higher education in Uganda.

## Quantitative results

The study sought to establish the dominant types of organizational structures employed in private chartered universities in Western Uganda and their relationship with academic staff performance. Descriptive statistics revealed that most respondents identified their universities as operating under mechanistic structures, characterized by clear hierarchies, centralized authority, formalized procedures, and functional departmentalization. Table 8 presents the correlation results between various structural dimensions and academic staff performance.

**Table 8.** Pearson’s linear correlation coefficient showing the relationship between organizational structure and academic staff performance.

Organizational structure dimension	Academic staff performance (r)	Sig. (2-tailed)	Interpretation
**Hierarchy & Chain of Command**	0.62	<0.01	Strong positive correlation
**Departmentalization**	0.57	<0.01	Moderate positive correlation
**Centralization & Decentralization**	0.68	<0.01	Strong positive correlation
**Formalization**	0.60	<0.01	Strong positive correlation
**Overall Organizational Structure**	**0.65**	**<0.01**	**Strong positive correlation**

The results in Table 8 indicate that all dimensions of organizational structure have a positive and statistically significant relationship with academic staff performance. Notably, centralization and decentralization exhibited the strongest correlation (r = 0.68, p < 0.01), suggesting that effective performance is fostered when decision-making balances top-down authority with departmental autonomy. Similarly, hierarchy and chain of command (r = 0.62, p < 0.01) and formalization (r = 0.60, p < 0.01) showed strong positive correlations, indicating that clarity in reporting lines and adherence to established policies contribute to improved teaching, research, and administrative performance. Departmentalization, though slightly lower (r = 0.57, p < 0.01), still demonstrated a significant positive influence, highlighting the value of organized faculty structures in supporting specialization and teamwork.

To further verify the differences among these structural elements, ANOVA was conducted, as shown in Table 9.

**Table 9.** ANOVA showing significant differences in organizational structure and academic staff performance.

Source	SS	df	MS	F	p
**Between Groups**	25.42	3	8.47	8.76	<.01
**Within Groups**	189.42	196	0.97		
**Total**	214.84	199			

The ANOVA results reveal statistically significant differences in academic staff performance across the different dimensions of organizational structure (F(3,196) = 8.76, p < .01). This implies that not all structural elements influence performance equally. For example, centralization and formalization tend to yield higher levels of accountability and efficiency, while departmentalization contributes to collaboration and specialization but may not directly translate into higher performance outcomes. This suggests that an optimal structure requires a balance between control and flexibility, allowing departments to operate autonomously while remaining aligned with institutional objectives.

To further understand predictive power, regression analysis was performed ( Table 10).

**Table 10.** Regression analysis predicting academic staff performance from organizational structure.

Predictor	B	SE B	β	t	p
Hierarchy & Chain of Command	0.28	0.07	0.23	4.00	<.001
Departmentalization	0.21	0.06	0.17	3.50	<.01
Centralization & Decentralization	0.31	0.08	0.26	4.25	<.001
Formalization	0.25	0.07	0.20	3.57	<.001

R ^2^ = 0.38, F = 28.90, p < .001.

The regression results show that organizational structure significantly predicts academic staff performance, explaining 38% of the variance (R ^2^ = 0.38, F = 28.90, p < .001). Among the predictors, centralization and decentralization (β = 0.26, p < .001) emerged as the strongest determinant of performance, underscoring the importance of shared decision-making and balanced authority. Hierarchy and chain of command (β = 0.23, p < .001) also play a major role by clarifying accountability, while formalization (β = 0.20, p < .001) enhances procedural fairness and consistency. Although departmentalization (β = 0.17, p < .01) had a smaller effect, it still positively contributed by promoting collaboration and academic specialization.

These quantitative results collectively indicate that universities with clear hierarchies, structured departments, and balanced centralization tend to achieve higher staff performance, provided these mechanisms are implemented with fairness and inclusivity.

## Qualitative results (Supporting evidence)

The qualitative interviews provided deeper understanding of how these structural features operate in practice. Deans generally acknowledged that hierarchical structures foster professionalism, order, and accountability, but they also expressed concerns about rigidity and limited participation. For instance, Dean 1 stated, “The relationship is generally cordial, though sometimes it feels supervisory rather than collaborative” (DU PT1-251), while Dean 6 observed that “sometimes the process feels top-down rather than participatory” (DU PT6-285). Such reflections align with Turyamureeba and Atwebembeire (2020), who caution that mechanistic structures in universities often suppress innovation and restrict collegiality.

Conversely, other Deans highlighted that formalized procedures and clear hierarchies can enhance coordination and performance accountability. Dean 2 emphasized, “I have a respectful and professional relationship with my supervisors; we communicate openly” (DU PT2-258), while Dean 4 added, “We maintain a formal yet supportive relationship that encourages improvement” (DU PT4-272). These perspectives confirm the positive correlation between formalization and performance found in quantitative results (r = 0.60, p < 0.01).

Moreover, discussions about centralization and decentralization revealed a mixed perception. Dean 4 commented that “It helps align staff goals with institutional priorities” (DU PT4-276), emphasizing that central coordination ensures consistency and alignment with institutional vision. However, Dean 9 warned that “inconsistent application undermines trust” (DU PT9-281), echoing Silaji et al. (2023), who found that fairness and procedural consistency are essential for maintaining staff motivation under structured systems.

When discussing career progression and job security, Deans acknowledged that structural clarity enhances accountability and recognition. Dean 2 remarked, “It directly affects promotions and contract renewals” (DU PT2-284), while Dean 4 stated, “It motivates me to perform better for advancement” (DU PT4-286). Nonetheless, concerns about favoritism were evident, with Dean 9 cautioning, “Favoritism can distort outcomes” (DU PT9-291). This qualitative insight supports Marisa and Oigo (2018), who argue that equity and fairness within formalized structures are crucial for sustaining trust and performance motivation.

This echoes Adams and Wang (2020), who found that excessive monitoring and administrative pressure contribute to academic staff burnout in private universities.

Similarly, Adekoya and Ajagbe (2021) noted that decentralised structures improve staff participation and accountability in Nigerian private universities.

Which also corresponds with Alam et al. (2021), who found that e-learning technologies promote sustainable academic performance through enhanced digital monitoring.

Resource constraints emerged as another significant theme moderating the effectiveness of structural systems. Dean 1 admitted, “Support exists, but resources like research funding are limited” (DU PT1-293), while Dean 8 lamented, “Support is inadequate, especially for research and publications” (DU PT8-300). These findings qualify the quantitative results by showing that even when structures are well-defined, insufficient institutional resources hinder staff performance a concern also echoed by Eze et al. (2024) and Rwothumio & Amwine (2021a), who found that institutional underfunding undermines the functionality of organizational structures in African universities. These findings suggest that academic staff engagement and collaboration enhance productivity, which aligns with Abdullahi et al. (2023), who found that knowledge sharing behavior significantly predicts staff performance in Malaysian private universities.

Similar patterns were observed by Mugizi et al. (2019d) in Ugandan private universities.

### Integrated discussion

By integrating both data sets, it becomes evident that organizational structure strongly influences academic staff performance in private chartered universities in Western Uganda. Quantitative results established statistically significant relationships across all structural dimensions (r = 0.65, p < .01), while qualitative evidence explained the contextual dynamics behind these associations. In particular, centralized but participatory structures appear most effective because they provide guidance without suppressing autonomy.

These findings are consistent with Silaji et al. (2025), who demonstrated that transparent and balanced structural systems promote accountability and performance in higher education. Similarly, Latif et al. (2024) argue that universities adopting hybrid structures combining hierarchical control with collaborative flexibility tend to achieve superior academic outcomes. Conversely, Atwebembeire and Malunda (2019) note that rigid mechanistic structures reduce adaptability, innovation, and morale.

### Demographic Characteristics of the Respondents for Quantitative Data of HOD and Lecturers (Academic Staff)

This section presents the demographic profile of the Lecturers and HOD (academic staff) who participated in the study. Variables analyzed include gender, academic qualifications, years of experience, and faculty affiliation. The purpose of this analysis was to provide context to the findings by understanding the characteristics of the respondents. And the data on these background characteristics is presented in Table 11 below.

**Table 11.** Background characteristics of the HOD and Lecturers.

Item	Category	Frequency	Percentage (%)
Gender	Male	196	62.2
	Female	119	37.8
Age Group	Below 30 years	20	6.50
	30–40 years	182	57.80
	40–50 years	93	29.50
	50 years and above	20	6.20
	Total	315	100.00
Highest Level of Education	Bachelor's Degree	50	15.80
	Master's Degree	188	59.80
	PhD	77	24.40
	Total	315	100.00
Position in the University	Teaching Assistant	58	18.40
	Assistant Lecturer	149	47.40
	Lecturer	56	17.60
	Senior Lecturer	31	9.80
	Associate Professor	13	4.10
	Professor	8	2.60
	Total	315	100.00
Years of Teaching Experience	Below 5 years	95	30.10
	5–10 years	96	30.60
	11–15 years	63	20.20
	16–20 years	42	13.20
	Over 20 years	19	6.00
	Total	315	100.00
Field of Specialty	Biomedicals	37	11.70
	Education	142	45.10
	Information Technology	18	5.70
	Pharmacy	10	3.10
	Engineering	54	17.10
	Business and Management	17	5.40
	Clinicals	13	4.20
	Nursing	17	5.30
	Agriculture	8	2.5
	Total	315	100.00

Table 11 above, show the analysis of the gender category revealed that the majority of the respondents were male (62.2%), while females comprised 37.8% of the sample. This means that the gender distribution shows a higher representation of male respondents. However, views were representative across both gender groups, indicating adequate gender inclusion and balance within the university.

The results regarding the age groups of the academic staff showed that a small percentage of the sample is 30 years old (6.5%), while, the largest group, representing more than half of the respondents is 30-40 years old (57.8%), followed by 29.5% that were of age between 40-50 years. The smallest respondents are 50 years and above (6.2%). The presence of academic staff above 50 years might indicate the institution’s inclusivity in hiring experienced educators. These results demonstrate that academic staff from various age groups participated in the study. As a result, the opinions expressed represented the opinions of academic staff members across a range of age groups, resulting in data that could be used for generalization.

Statistics on the highest educational level attained revealed that a greater proportion of the academic staff (59.8%) have a master’s degree, followed by around a quarter of the respondents holding a PhD (24.4%), and the percentage of responders with a bachelor’s degree is just 15.8%. The data suggests that most faculty members are well-qualified, with master’s and PhD holders constituting the majority. This indicates a highly educated academic environment; therefore, the views were representative of staff from different levels of education.

In the same vein, the results regarding positions held at the university indicated that the majority, 47.4%, were assistant lecturers, followed by (18.4%) who were teaching assistants, (17.6 %) were lecturers, senior lecturers have the percentage of (9.8%), with associate professors/professors (2.6%). This suggested that views were representative of the different positions held in the university. The information regarding years of teaching experience reveals the larger percentage (30.6%) were academic staff that have worked below 5-10 years in the university, followed by (30.1%) below 5 years (10%), 11-15 years of teaching experience have the percentage of (20.2%), while 16-20 years were (13.2%).

A small percentage has over 20 years of experience (6.0%). The teaching staff appears to have a balanced mix of both early-career and mid-career faculty members. Hence, the views presented encompass the views of academic staff with diverse years of experience, thereby offering data that can be generalized.

The distribution of academic staff across faculties/schools is as follows: The largest group specializes in education (45.1%), Engineering (17.1 %), followed by Biomedical (11.7%), Pharmacy (3.1%), Information Technology (5.7%), Business and Management (5.4%). The faculty specialization is predominantly in the field of education, followed by engineering. This indicates a strong presence of education-focused programs at the university, with a reasonable representation from other technical fields such as biomedical and engineering disciplines.

### Demographic Characteristics of the Respondents for Qualitative Data (Faculty Deans)

The demographic table summarizes the academic staff who participated in answering interview guide in the study.

### Interpretation of the Demographic Table 12

**Table 12.** Demographic Characteristics of Faculty Deans (N=10).

Participant ID	Position	Highest qualification	Specialization	Years in current position	Total years of experience
PT1	Dean	PhD	Biomedicals	Since 2012	Since 2007
PT2	Dean	PhD	Education	5 years	17 years
PT3	Dean	PhD	Information Technology	5 years	15 years
PT4	Dean	PhD	Pharmacy	2 years, 6 months	13–14 years
PT5	Dean	PhD	Engineering	4 years	9 years
PT6	Dean	PhD	Business and Management	4 years	9 years
PT7	Dean	PhD	Clinicals	1 year, 7 months	24 years
PT8	Dean	PhD	Nursing	Since Feb 5, 2023	18 years
PT9	Dean	PhD	Agriculture	Since April	17 years
PT10	Dean	PhD		4 years	6 years

Although 15 deans were initially selected for participation in this study, only 10 ultimately took part in the interview. The reduced participation was due to factors such as scheduling conflicts, limited availability, and non-responsiveness from some of the selected individuals. Despite this, the responses from the 10 participating deans provided rich and relevant insights that were sufficient to meet the objectives of the study.

Therefore, the above Table 12 provides an overview of the demographic characteristics of the Deans who participated in the interview. It provides key insights into their qualifications, experience, and faculty distribution, aligning with the study’s objective of understanding the organizational structure, performance monitoring, and academic staff performance in private chartered universities in Western Uganda Findings indicate that all faculty heads hold PhDs, demonstrating a strong emphasis on academic qualifications in leadership roles. The tenure in current positions varies, with some deans having served for over five years, while others are relatively new, suggesting a balance between leadership stability and transition. The experience levels range from six to twenty-four years (6-24yrs) showing diversity in leadership maturity. Faculty representation spans Business, Education, Health sciences, Engineering, and Technology, ensuring broad institutional perspectives on performance monitoring and academic structures. These findings provide context for analyzing how organizational structure and performance monitoring impact academic staff performance, highlighting the need for effective leadership, professional development support, and balanced workload distribution to enhance institutional efficiency.

## Conclusion

This study concludes that private chartered universities in Western Uganda predominantly use hierarchical and functional organizational structures, with some institutions adopting matrix structures to promote flexibility. Organizational structure has a direct impact on academic staff performance, influencing communication, role clarity, and collaboration. Although hierarchical and functional structures are effective for administrative control and specialization, matrix structures may foster innovation and interdisciplinary work, although they require careful management to avoid role ambiguity.

This supports Adhikari and Shrestha (2023), who emphasized knowledge management initiatives in universities as key to achieving SDG 4.7 through inclusive and equitable education. 

Overall, both the quantitative and qualitative results confirm that organizational structure significantly predicts academic staff performance. While hierarchy, formalization, and centralization ensure order and efficiency, over-centralization can suppress initiative. Therefore, a balanced structural framework one that combines clear accountability, departmental autonomy, and adequate resource support is essential for enhancing performance and institutional effectiveness in private chartered universities in Western Uganda.

### Limitations and future research

This study was limited by its cross-sectional design, which restricts causal interpretation. The sample was drawn from private chartered universities in Western Uganda, limiting generalizability to other regions or public institutions. Future research could employ longitudinal designs and include comparative analyses across multiple contexts to examine structural dynamics over time. In addition, integrating organizational culture and leadership style variables could enrich the understanding of how structure interacts with other institutional factors.

### Recommendations

Based on the findings, the study recommends the following:

Universities should review their organizational structures to ensure that they align with academic staff needs and institutional goals.

Adopting more flexible matrix-based structures in research departments to foster innovation and collaboration.

Provide training for academic staff and leadership to navigate complex organizational structures and improve communication and collaboration.

## Data Availability

OSF - Examining types of Organizational Structure in Private Chartered Universities in Western Uganda and their Impact on Academic Staff Performance, https://doi.org/10.17605/OSF.IO/YKDV5 ( Silaji et al., 2025). This project contains following dataset:
- Dr. Silaji. Sav OSF - Examining types of Organizational Structure in Private Chartered Universities in Western Uganda and their Impact on Academic Staff Performance, https://doi.org/10.17605/OSF.IO/YKDV5 ( Silaji et al., 2025). This project contains the following extended data:
- Interview responses.docx Data are available under the terms of the Creative Commons Attribution 4.0 International license (CC-BY 4.0).
