# Supplementary material for: Examining types of Organizational Structure in Private Chartered Universities in Western Uganda and their Impact on Academic Staff Performance
Source: F1000Res. 2026 Mar 11;14:690. Originally published 2025 Jul 14. [Version 3] doi: 10.12688/f1000research.164151.3 (PMC12877467; doi:10.12688/f1000research.164151.3)
Supplement: Peer review history

# Reviewer report: Reviewer response for version 3

**DOI:** 10.5256/f1000research.196362.r466699

**Published:** 2026-04-02

**Authors:** Shahid Rafiq

**Affiliations:** 1 Emerson UniversityMultan, Multan, Pakistan

The authors have adequately addressed the concerns raised in my previous report, and the revisions have improved the clarity and overall quality of the manuscript.

Is the work clearly and accurately presented and does it cite the current literature?

Partly

If applicable, is the statistical analysis and its interpretation appropriate?

Partly

Are all the source data underlying the results available to ensure full reproducibility?

Partly

Is the study design appropriate and is the work technically sound?

Partly

Are the conclusions drawn adequately supported by the results?

Yes

Are sufficient details of methods and analysis provided to allow replication by others?

Partly

Reviewer Expertise:

NA

I confirm that I have read this submission and believe that I have an appropriate level of expertise to confirm that it is of an acceptable scientific standard.

# Reviewer report: Reviewer response for version 2

**DOI:** 10.5256/f1000research.190541.r451592

**Published:** 2026-02-05

**Authors:** Jacqueline Y. Sánchez-García, Juan E. Núñez-Ríos

**Affiliations:** 1 Guadalajara Universidad Panamericana, Zapopan, Mexico; 2 Business School, Universidad Panamericana (Ringgold ID: 431677), Mexico City, Mexico

I appreciate the invitation to review this work and, with all due respect to the authors, I recognize that the paper has an organized structure that facilitates overall understanding of the study.

 With regard to the objectives, it is considered that consistent efforts are being made to achieve them. However, I would like to share some observations with a view to contributing constructively to your work:

- With regard to the theoretical approach, it should be clarified how the components of expectation theory are included and substantiated in the research, since they are not explicitly measured in either the quantitative or qualitative parts. It is considered that from the outset it should be specified that it serves as an interpretative framework rather than a theoretical foundation or empirical scope.
- Methodologically, it is recommended to justify why inferential statistical techniques were chosen and methods such as factor analysis, structural equation models, or nonparametric techniques were not considered, given that the data comes from the use of the Likert scale.
- The study identifies the dimensions of Fayol's theory that are predominant in private universities and demonstrates significant relationships between organizational structure and academic staff performance. Therefore, the study reviews these dimensions rather than reviewing the types of structures. For this reason, the authors are respectfully invited to clarify the conceptual use of the term “types of structures.” From a methodological perspective, both the instrument and the analyses used identify the structural configuration rather than the types of structures, and if that was the scope, then the study could be limited.

Is the work clearly and accurately presented and does it cite the current literature?

Partly

If applicable, is the statistical analysis and its interpretation appropriate?

Yes

Are all the source data underlying the results available to ensure full reproducibility?

Partly

Is the study design appropriate and is the work technically sound?

Yes

Are the conclusions drawn adequately supported by the results?

Partly

Are sufficient details of methods and analysis provided to allow replication by others?

Partly

Reviewer Expertise:

organizational theory and organizational design

We confirm that we have read this submission and believe that we have an appropriate level of expertise to confirm that it is of an acceptable scientific standard, however we have significant reservations, as outlined above.

# Response

**Published:** 2026-02-06

**Authors:** Turyamureeba silaji

**Affiliations:** Foundations, Kampala International University - Western Campus, Bushenyi, Western Region, Uganda

We sincerely thank the reviewer for the careful reading of our manuscript and for the constructive and insightful comments. We appreciate the positive remarks regarding the organisation and clarity of the paper. Below, we respond to each comment point by point and indicate how the manuscript has been revised accordingly.

**Comment 1: Clarification on the use of Expectancy Theory**

*Reviewer’s comment:*

 The reviewer notes that expectancy theory is not explicitly measured in the quantitative or qualitative components of the study and recommends clarifying whether it is used as an interpretative framework rather than as a theoretical foundation with empirical scope.

**Response:**

 We appreciate this important observation. We agree with the reviewer that expectancy theory was not operationalised through direct measurement of its core components (expectancy, instrumentality, and valence) in either the quantitative or qualitative phases of the study.

 In response, the manuscript has been revised to explicitly position expectancy theory as an interpretative framework, rather than as a theory subjected to empirical testing. The theory is now used to interpret academic staff motivation and performance behaviours, particularly in explaining how organisational structure and performance monitoring practices shape staff expectations, perceived rewards, and effort–performance linkages.

 This clarification has been incorporated in the theoretical framework section, where expectancy theory is presented as a supportive lens for interpretation, complementing organisational structure and performance management theories, rather than serving as a direct analytical or measurement model.

**Comment 2: Justification for inferential statistical techniques**

*Reviewer’s comment:*

 The reviewer recommends justifying the choice of inferential statistical techniques and explaining why methods such as factor analysis, structural equation modelling, or nonparametric techniques were not employed, given the use of Likert-scale data.

**Response:**

 We thank the reviewer for this methodological suggestion. The manuscript has been revised to provide a clearer justification for the choice of inferential statistical techniques.

 The study employed correlation and regression analyses because the primary objective was to examine relationships and predictive effects between organisational structure, performance monitoring, and academic staff performance, rather than to validate latent constructs or test complex causal pathways.

 Factor analysis and structural equation modelling were not adopted because:

1. The study was not designed to develop or validate new measurement scales, as the constructs were adapted from established literature.
2. The sample size and research objectives were more suited to relationship testing than to complex structural modelling.
3. The Likert-scale data were treated as interval-level measurements, consistent with common practice in social science research, thus justifying the use of parametric techniques.

These clarifications have been added to the **methodology section**, specifically under data analysis procedures.

**Comment 3: Conceptual use of “types of organisational structures”**

*Reviewer’s comment:*

 The reviewer observes that the study identifies and analyses dimensions derived from Fayol’s theory rather than organisational structure types, and invites the authors to clarify the conceptual use of the term “types of structures.”

**Response:**

 We are grateful for this important conceptual clarification. We agree with the reviewer that the study empirically examines structural dimensions such as hierarchy, centralisation, formalisation, and departmentalisation rather than directly measuring predefined organisational structure types.

 In response, the manuscript has been revised to clarify that organisational structure types (mechanistic and organic) are inferred from the configuration of structural dimensions, rather than being measured as discrete categories. The term “types of organisational structure” is now explicitly used in a conceptual and interpretative sense, based on the dominant patterns emerging from the analysed dimensions.

 Accordingly:

- The conceptual framework now clearly shows dimensions leading to inferred structural types.
- The results and discussion sections emphasise “dominant structural characteristics” and “structural configurations” rather than treating structure types as directly observed variables.

This revision ensures conceptual precision and alignment between the study’s instruments, analyses, and stated scope.

 Conclusion

 We sincerely thank the reviewer for these valuable comments, which have significantly strengthened the theoretical clarity, methodological justification, and conceptual precision of the manuscript. We believe the revisions have improved the overall rigour and contribution of the study.

 Kind regards

 Turyamureeba Silaji

 Corresponding author

# Reviewer report: Reviewer response for version 2

**DOI:** 10.5256/f1000research.190541.r448164

**Published:** 2026-01-30

**Authors:** Issah Iddrisu

**Affiliations:** 1 University for Development Studies, Tamale, Ghana

The manuscript has been thoroughly revised and now meets the expected standards for indexing in *F1000Research*. The authors have responded comprehensively to all previous feedback, resulting in a more robust, transparent, and impactful study.

Is the work clearly and accurately presented and does it cite the current literature?

Partly

If applicable, is the statistical analysis and its interpretation appropriate?

Yes

Are all the source data underlying the results available to ensure full reproducibility?

Yes

Is the study design appropriate and is the work technically sound?

Yes

Are the conclusions drawn adequately supported by the results?

Yes

Are sufficient details of methods and analysis provided to allow replication by others?

Partly

Reviewer Expertise:

MANAGEMENT AND ORGANISATIONAL STUDIES

I confirm that I have read this submission and believe that I have an appropriate level of expertise to confirm that it is of an acceptable scientific standard.

# Response

**Published:** 2026-01-30

**Authors:** Turyamureeba silaji

**Affiliations:** Foundations, Kampala International University - Western Campus, Bushenyi, Western Region, Uganda

Dear Dr. Issah Idris in your full Honors

 We sincerely thank you for your time and thoughtful review of our manuscript. We are grateful for your careful assessment and for acknowledging the improvements made in response to the previous feedback.

 Your comments and overall evaluation were invaluable in strengthening the clarity, transparency, and robustness of the study. We truly appreciate your expertise and the constructive role you played in helping our work meet the standards required for indexing in F1000Research.

 Thank you once again for your professionalism and support.

 Kind regards,

 Turyamureeba silaji 

 On behalf of the Authors

# Reviewer report: Reviewer response for version 1

**DOI:** 10.5256/f1000research.180615.r399615

**Published:** 2025-10-28

**Authors:** Issah Iddrisu

**Affiliations:** 1 University for Development Studies, Tamale, Ghana

**Reviewer Report**

**1. Is the work clearly and accurately presented and does it cite the current literature?**

**Partly.**

- Add a clear statement of the research gap and contribution at the end of the literature review.
- Reduce redundant citations and improve transitions between paragraphs.
- Consider synthesizing studies thematically (e.g., hierarchical vs. functional vs. matrix) rather than listing individually.

**2. Is the study design appropriate and is the work technically sound?**

**Mostly yes, but some methodological clarifications are needed.**

- Include sampling rationale, inclusion/exclusion criteria, and demographic characteristics of respondents.
- Report reliability coefficients (Cronbach’s alpha or composite reliability) for each construct.
- Strengthen the integration of qualitative and quantitative results in the discussion.

**3. Are sufficient details of methods and analysis provided to allow replication by others?**

**Partly.**

- Add a table summarizing constructs, items, and measurement sources.
- Include a brief subsection on “Data analysis procedures” to distinguish between quantitative (SPSS, regression, correlation) and qualitative (NVivo, thematic analysis) steps.
- Discuss ethical clearance more succinctly—keep key approval numbers but reduce repetition.

**4. Is the statistical analysis and its interpretation appropriate?**

**Largely appropriate, but could be strengthened.**

- Expand the explanation of R² and F-statistics to interpret effect strength.
- Present a summary table for reliability, validity, and discriminant validity (if applicable).
- Use APA or F1000 statistical reporting standards consistently (e.g., *r* = .53, *p* < .01).

**5. Are the conclusions supported by the results?**

**Yes, but they could be more analytical and less descriptive.**

- Add a short subsection titled “Implications for Policy and Practice.”
- Discuss limitations (e.g., small sample, cross-sectional design) and suggest areas for future research.
- End with a concise summary paragraph reinforcing the main contribution.

**6. Is the language and style appropriate?**

- Edit for brevity and clarity, especially in the literature review and methodology.
- Simplify tables—retain essential figures and move detailed statistics to supplementary materials if possible.
- Ensure consistent tense (past tense for results, present for discussion).

**7. Overall Recommendation**

**Status:** *Approve with major revisions.*

 The paper addresses a relevant and underexplored issue in higher education management in Uganda. Its theoretical grounding and mixed-methods approach are strong, but the paper needs revision to improve clarity, methodological transparency, and analytical depth.

Is the work clearly and accurately presented and does it cite the current literature?

Partly

If applicable, is the statistical analysis and its interpretation appropriate?

Yes

Are all the source data underlying the results available to ensure full reproducibility?

Yes

Is the study design appropriate and is the work technically sound?

Yes

Are the conclusions drawn adequately supported by the results?

Yes

Are sufficient details of methods and analysis provided to allow replication by others?

Partly

Reviewer Expertise:

MANAGEMENT AND ORGANISATIONAL STUDIES

I confirm that I have read this submission and believe that I have an appropriate level of expertise to confirm that it is of an acceptable scientific standard, however I have significant reservations, as outlined above.

# Response

**Published:** 2025-10-29

**Authors:** Turyamureeba silaji

**Affiliations:** Foundations, Kampala International University - Western Campus, Bushenyi, Western Region, Uganda

**Response to Reviewer** **2** **Comments**

 We sincerely thank the reviewer for the constructive and insightful comments on our manuscript. We have carefully considered each point and revised the manuscript accordingly. Below is a point-by-point response to the comments:

**Reviewer Comment 1:** *Is the work clearly and accurately presented and does it cite the current literature?*

**Response:** Partly.

- We have added a clear statement of the research gap and the study’s contribution at the end of the literature review to enhance clarity.
- Redundant citations have been reduced, and transitions between paragraphs have been improved for better flow.
- Studies are now synthesized thematically (hierarchical, functional, and matrix structures) rather than listed individually.

**Reviewer Comment 2:** *Is the study design appropriate and is the work technically sound?*

**Response:** Mostly yes.

- We have included the sampling rationale and clearly defined inclusion/exclusion criteria.
- Demographic characteristics of respondents have been presented in a new table for transparency.
- Reliability coefficients (Cronbach’s alpha) for each construct have been added.
- The discussion now integrates qualitative and quantitative results more cohesively, highlighting how each informs the other.

**Reviewer Comment 3:** *Are sufficient details of methods and analysis provided to allow replication by others?*

**Response:** Partly.

- A table summarizing constructs, items, and measurement sources has been included.
- A new subsection “Data Analysis Procedures” distinguishes quantitative (SPSS, regression, correlation) and qualitative (NVivo, thematic analysis) steps.
- Ethical clearance details have been retained succinctly with key approval numbers and reduced repetition.

**Reviewer Comment 4:** *Is the statistical analysis and its interpretation appropriate?*

**Response:** Largely appropriate.

- The explanation of R² and F-statistics has been expanded to interpret effect size and model strength.
- A summary table for reliability, validity, and discriminant validity has been added.
- All statistical reporting now follows APA/F1000 standards consistently (e.g., r = .53, p < .01).

**Reviewer Comment 5:** *Are the conclusions supported by the results?*

**Response:** Yes.

- A new subsection, “Implications for Policy and Practice,” has been added to highlight actionable insights.
- Limitations (e.g., small sample size, cross-sectional design) are now discussed, with suggestions for future research.
- The manuscript ends with a concise summary paragraph reinforcing the main contribution.

**Reviewer Comment 6:** *Is the language and style appropriate?*

**Response:**

- The manuscript has been edited for brevity and clarity, particularly in the literature review and methodology sections.
- Tables have been simplified, retaining only essential figures; detailed statistics have been moved to supplementary materials.
- Tense usage has been standardized (past tense for results, present tense for discussion).

**Reviewer Comment 7:** *Overall Recommendation*

**Response:** We appreciate the reviewer’s acknowledgment of the relevance and strength of our study. All suggested revisions have been incorporated to improve clarity, methodological transparency, and analytical depth.

 We believe these revisions have significantly strengthened the manuscript, and we thank the reviewer again for their valuable feedback.

 Yours faithfully

 Turyamureeba Silaji

 Corresponding Author

# Reviewer report: Reviewer response for version 1

**DOI:** 10.5256/f1000research.180615.r422941

**Published:** 2025-10-28

**Authors:** Shahid Rafiq

**Affiliations:** 1 Emerson UniversityMultan, Multan, Pakistan

The article *“Examining types of Organizational Structure in Private Chartered Universities in Western Uganda and their Impact on Academic Staff Performance”* investigates how different organizational structures influence staff performance in private universities. The study draws on Henri Fayol’s Administrative Management Theory and Vroom’s Expectancy Theory and applies a mixed-methods design. It combines quantitative data from 186 academic staff members with qualitative insights from interviews with 10 academic deans. The findings show that hierarchical and functional structures are the most common, with centralization playing a dominant role. Statistical analysis reveals that organizational structure accounts for 26.2% of the variance in academic staff performance, and qualitative data reinforce the view that rigid structures can restrict autonomy and innovation. The authors conclude that more adaptive and participatory structures could improve staff performance and institutional effectiveness.

 The article is clearly structured and mostly well written. It provides a solid theoretical foundation and cites both classical and recent literature. However, some sections are overly descriptive, and several references are repeated unnecessarily. A more focused literature review would make the argument clearer and more cohesive. While the study design is appropriate for the research objectives, some important methodological details are missing. The paper does not explain how participants were selected, how the survey instrument was validated, or how qualitative data were analyzed. These are critical elements that should be addressed to strengthen the technical rigor of the study.

 The methods section offers an overview of data collection and analysis, but not enough information to allow replication by other researchers. The authors should include more precise details on sampling procedures, provide or reference the survey instrument and interview guide, and describe how data were coded and analyzed. The statistical analysis itself is appropriate, with the use of descriptive statistics, correlation, and regression. However, more transparency is needed in reporting results. Important details such as regression coefficients, confidence intervals, assumptions testing, and effect sizes are missing. Including these would strengthen the credibility and reproducibility of the findings.

 Another limitation is the lack of access to the underlying dataset. The paper does not include a clear data availability statement or links to supporting materials. Providing anonymized raw data or explaining why it cannot be shared would be necessary to ensure transparency. Despite these methodological gaps, the conclusions are well supported by the data. The discussion appropriately connects the results to existing literature and highlights how organizational structure affects academic staff motivation, autonomy, and performance.

 In terms of presentation, the article is easy to follow and well organized. The tables are clear and the narrative flows logically. Some sections would benefit from more concise writing and the removal of repetitive phrases. Minor grammatical improvements would also enhance readability. Overall, this is a relevant and timely study with clear implications for university governance and staff performance in the Ugandan context.

 To make the article scientifically sound, the authors should address four key areas: first, provide fuller methodological details, including sampling procedures, instrument development, and qualitative analysis methods; second, strengthen statistical reporting with more technical transparency; third, clarify or include data availability to support reproducibility; and fourth, tighten the literature review to remove unnecessary repetition. Addressing these points would significantly enhance the rigor, clarity, and impact of the work

Is the work clearly and accurately presented and does it cite the current literature?

Partly

If applicable, is the statistical analysis and its interpretation appropriate?

Partly

Are all the source data underlying the results available to ensure full reproducibility?

Partly

Is the study design appropriate and is the work technically sound?

Partly

Are the conclusions drawn adequately supported by the results?

Yes

Are sufficient details of methods and analysis provided to allow replication by others?

Partly

Reviewer Expertise:

Educational Leadership, Higher Education Management, and Organizational Studies.

I confirm that I have read this submission and believe that I have an appropriate level of expertise to confirm that it is of an acceptable scientific standard, however I have significant reservations, as outlined above.

# Response

**Published:** 2025-10-28

**Authors:** Turyamureeba silaji

**Affiliations:** Foundations, Kampala International University - Western Campus, Bushenyi, Western Region, Uganda

**Response to Peer Reviewer Comments**

**Manuscript Title: *Examining Types of Organisational Structure in Private Chartered Universities in Western Uganda and Their Impact on Academic Staff Performance***

**Journal: *F1000Research***

 We sincerely thank the reviewer for the detailed and constructive feedback provided on our manuscript. The comments were insightful and have significantly improved the overall quality, clarity, and rigor of the paper. Below, we provide a point-by-point response outlining how each issue has been addressed in the revised version.

**1. General Comment**

**Reviewer Comment:**

 The article is clearly structured and mostly well written. It provides a solid theoretical foundation and cites both classical and recent literature. However, some sections are overly descriptive, and several references are repeated unnecessarily. A more focused literature review would make the argument clearer and more cohesive.

**Response:**

 We appreciate this positive evaluation. The literature review section has been thoroughly revised to reduce redundancy and improve coherence. Repeated citations (e.g., multiple mentions of Atwebembeire et al., Mugizi et al., and Marisa & Oigo) have been streamlined, and the review now follows a thematic and critical format. Additionally, more recent and relevant literature has been incorporated (e.g., Silaji et al., 2023; Eze et al., 2024; Latif et al., 2024; Silaji et al., 2025) to enhance the argument and theoretical integration.

**Revision Made:**

 Section 2.0 *Literature Review* reorganized thematically around Hierarchy, Centralization, Formalization, and Departmentalization.

 Redundant citations removed; all references updated to APA 7 style.

 Added critical synthesis linking Fayol’s Administrative Theory and Vroom’s Expectancy Theory to organizational design and motivation outcomes.

**2. Methodological Detail**

**Reviewer Comment:**

 The paper does not explain how participants were selected, how the survey instrument was validated, or how qualitative data were analyzed. These are critical elements that should be addressed to strengthen the technical rigor of the study.

**Response:**

 We agree that methodological transparency is essential. Accordingly, the Methods section has been expanded substantially. We have detailed participant selection, instrument validation, and qualitative analysis procedures.

**Revisions Made:**

 Added a Sampling subsection describing the stratified random selection of 186 academic staff from four private chartered universities, and the purposive inclusion of 10 academic deans.

 Included a Survey Instrument subsection specifying that the questionnaire was adapted from validated tools (Mugizi et al., 2019; Atwebembeire & Malunda, 2019), reviewed by experts, and pre-tested with a Cronbach’s alpha of 0.86 for reliability.

 Added a Qualitative Data Analysis subsection, explaining that interviews were transcribed and analyzed thematically using Braun & Clarke’s (2006) six-step framework.

 Included ethical clearance details and informed consent procedures to demonstrate research integrity.

**3. Statistical Transparency**

**Reviewer Comment:**

 Important details such as regression coefficients, confidence intervals, assumptions testing, and effect sizes are missing. Including these would strengthen the credibility and reproducibility of the findings.

**Response:**

 We have now provided comprehensive statistical reporting to enhance transparency.

**Revisions Made:**

 Regression tables now include β coefficients, standard errors, t-values, and significance levels (p-values).

 Added information on assumptions testing (normality, multicollinearity, and homoscedasticity) and confirmed that all statistical assumptions were met.

 Reported R² values, F-statistics, and confidence intervals for regression models.

 Clarified that analyses were conducted using SPSS Version 26.

 This additional information ensures full replicability and statistical rigor in line with the reviewer’s recommendations.

**4. Data Availability and Transparency**

**Reviewer Comment:**

 Another limitation is the lack of access to the underlying dataset. The paper does not include a clear data availability statement or links to supporting materials.

**Response:**

 A Data Availability Statement has now been included in the final section of the article to comply with F1000Research’s transparency policy.

**Revisions Made:**

*“The anonymized dataset supporting the findings of this study is available from the corresponding author upon reasonable request.”*

 This ensures openness and reproducibility while maintaining participant confidentiality in compliance with institutional ethical guidelines.

**5. Discussion and Theoretical Integration**

**Reviewer Comment:**

 The discussion appropriately connects the results to existing literature and highlights how organizational structure affects academic staff motivation, autonomy, and performance.

**Response:**

 We thank the reviewer for this positive observation. The discussion section has been strengthened further to deepen theoretical integration and enhance the connection between findings and theories.

**Revisions Made:**

 Incorporated Henri Fayol’s and Vroom’s frameworks explicitly to interpret results.

 Used connecting phrases such as *“Consistent with Fayol’s principle of unity of command…”* and *“In line with Vroom’s expectancy framework, excessive centralisation may weaken motivation…”*.

 Integrated both quantitative results (r = 0.65, p < .01; β = 0.26, p < .001) and qualitative evidence (e.g., Dean 1–10 statements) to provide a richer interpretation.

 Cited supporting literature (Rwothumio & Amwine, 2021; Silaji et al., 2025; Eze et al., 2024) to situate findings in current research.

**6. Writing Style and Clarity**

**Reviewer Comment:**

 Some sections would benefit from more concise writing and the removal of repetitive phrases. Minor grammatical improvements would also enhance readability.

**Response:**

 We have carefully reviewed the manuscript to ensure conciseness, clarity, and grammatical accuracy.

**Revisions Made:**

 Removed redundant phrases such as *“the findings revealed that…”* and simplified transitions.

 Enhanced readability through clearer topic sentences and paragraph transitions.

 Conducted a professional grammar check to improve overall flow.

 Reduced overly descriptive explanations to make arguments more precise.

**7. Contribution and Relevance**

**Reviewer Comment:**

 Overall, this is a relevant and timely study with clear implications for university governance and staff performance in the Ugandan context.

**Response:**

 We appreciate this encouraging feedback. We have further emphasized the policy implications of the findings in the Conclusion and Recommendations section, highlighting how private universities can adopt hybrid structures that balance accountability with flexibility.

**Revisions Made:**

 Expanded recommendations on participatory leadership, bottom-up communication, and periodic structural audits.

 Linked findings to the Ministry of Education and Sports (Uganda) policy framework to demonstrate national relevance.

**Closing Statement**

 We are grateful for the reviewer’s constructive comments, which have helped us significantly improve the rigor, clarity, and scholarly contribution of our manuscript. We believe that the revised version fully addresses all the concerns raised and aligns with the high standards expected by *F1000Research*.

 We hope the reviewer will find this revised manuscript satisfactory for indexing consideration.

 Signed,

 Turyamureeba Silaji
